# Lattice Disorder in CuI Enables Filament‐Free Transparent Memristors

**DOI:** 10.1002/advs.78209

**Published:** 2026-10-11

**Authors:** Yuzhao Ouyang, Ke Qu, Haonan Wang, Jianquan Liu, Wei Li, Zhuoran Ji, Jialin Yang, Qiuxiang Zhu, Zhenzhong Yang, Xiaodong Tang, Hui Peng, Bobo Tian, Chungang Duan, Chang Yang

**Affiliations:** ^1^ Key Laboratory of Polar Materials and Devices (MOE) Department of Electronics School of Information and Electronic Engineering East China Normal University Shanghai China; ^2^ Shanghai Centre of Brain‐inspired Intelligent Materials and Devices East China Normal University Shanghai China; ^3^ Shanghai Transparent Semiconductor Technology Co., Ltd. Shanghai China; ^4^ Collaborative Innovation Center of Extreme Optics Shanxi University Taiyuan Shanxi China

**Keywords:** distributed ion migration, filament‐free switching, interfacial barrier modulation, neuromorphic electronics, P‐type transparent memristor

## Abstract

The realization of invisible neuromorphic electronics hinges on transparent memories that reconcile high optical transparency with stable analog tunability, which is a challenge unmet by conventional filamentary devices. Here, we introduce an alloyed Cu_1‐_ *_x_* Ag*_x_* I halide transparent semiconductor with dual ionic‐electronic character, which enables a filament‐free switching platform. Ag^+^ incorporation induces lattice distortions within the γ‐phase CuI matrix, expanding interstitial channels and lowering the migration barrier without phase transition. This activates room‐temperature ion migration to dynamically modulate the p‐type hole conductance. Asymmetric electrodes induce spatially distributed ion migration and interfacial modulation, avoiding localized metallic percolation. In situ TEM and EDS mapping confirm reversible Cu^+^/Ag^+^ redistribution without continuous conductive pathways. Pt/Cu_1‐_ *_x_* Ag*_x_* I/Ag devices exhibit > 80% transparency, low‐voltage readout, and > 2‐year retention. They emulate synaptic plasticity and achieve high‐accuracy handwritten digit recognition. Our findings establish a design strategy for transparent memristors, enabling reliable neuromorphic hardware via ionically mediated electronic transport.

## Graphical abstract

Transparent Cu_1‐_ *_x_* Ag*_x_* I thin‐film memristors rely on internal ion transport to produce reversible resistive‐switching effects. Direct elemental characterizations observe obvious Cu and Ag redistribution driven by applied bias, proving mobile ions drive the device's resistive‐switching responses. These devices achieve high on‐off ratios, and the constructed artificial neural network reaches 98 % accuracy in recognizing handwritten digit images.

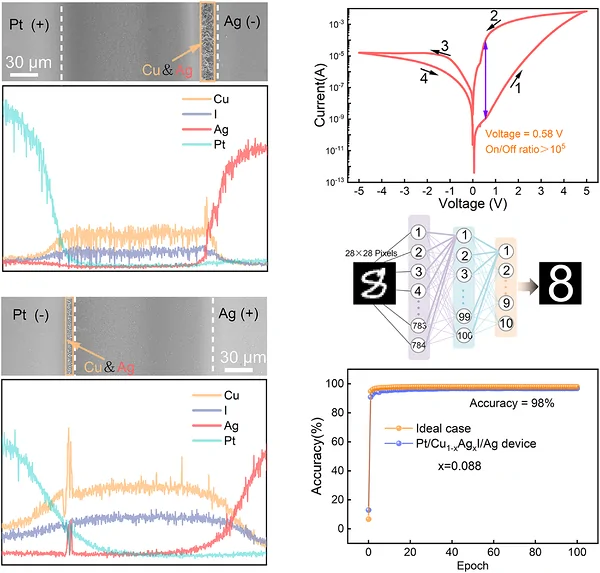

## 1 Introduction

The convergence of neuromorphic computing with invisible electronics, wearable systems, and biointerfaces demands a new class of high‐performance transparent memory, which is a critical building block that remains conspicuously absent from the materials library [1, 2, 3, 4, 5, 6, 7, 8]. Beyond visual unobtrusiveness, optical transparency preserves optical access to the underlying or overlying components, allowing electronic memory and neuromorphic functions to be integrated into systems where light transmission or optical readout is required. While significant progress has been made in n‐type transparent semiconductors (e.g., HfO*_x_*, WO*_x_*, IGZO) [9, 10, 11, 12, 13, 14], transparent p‐type semiconductors remain comparatively limited, particularly for applications requiring stable and continuously tunable electronic states. Conventional resistive‐switching approaches in transparent materials often rely on localized conductive‐filament (CF) formation, which can introduce stochastic switching and limit analog programmability [15, 16, 17, 18]. Recent advances suggest that moving away from localized filaments toward spatially distributed ionic processes may offer a viable pathway [19, 20, 21, 22, 23]. Importantly, filament‐free switching is not intrinsically restricted to p‐type materials; rather, it provides a general route toward more spatially distributed and reproducible conductance modulation. In this work, we focus on the p‐type transparent semiconductor Copper iodide (CuI) as a representative materials platform to address this specific materials challenge. CuI represents a prototypical transparent p‐type semiconductor, uniquely combining high visible‐light transmittance (> 80%) with exceptional hole mobility (> 40 cm^2^V^−1^s^−1^ in bulk) [24, 25, 26, 27, 28]. While CuI hosts intrinsically mobile Cu^+^ ions, its ionic conductivity is predominantly activated only in the high‐temperature *α‐* or *β*‐phase, rendering the room‐temperature *γ*‐phase predominantly hole‐conducting but ionically latent [25, 29, 30]. To address this materials challenge, we introduce an alloyed Cu_1‐_ *_x_* Ag*_x_* I solid solution. Ag^+^ incorporation induces targeted lattice disorder within the γ‑CuI matrix, expanding ion‐transport pathways and facilitating ionic transport. This transforms the material from a hole‐transport‐dominated semiconductor into an ionically active medium, wherein distributed ionic modulation supersedes localized filament formation. By integrating this optimized platform with asymmetric electrodes, we realize a filament‐free switching mechanism based on distributed ionic redistribution and interfacial barrier modulation. This approach yields homogeneous Cu^+^/Ag^+^ redistribution, which can be proved by in situ high‐resolution transmission electron microscopy (TEM). Notably, this mechanism manifests as a hallmark reversal of conventional switching polarity, where resistive switching is triggered by a positive bias on the nominally inert electrode. Our findings establish a general materials design strategy for transparent memristors, wherein the rational tuning of lattice topology unlocks ion‐electron synergy for reliable, invisible neuromorphic hardware.

## 2 Results

### 2.1 Structural, Optical, and Memristive Properties of Cu_1‐_ *_x_* Ag*_x_* I Devices

The structural, optical, and electrical characteristics of sputtered Cu_1‐_ *_x_* Ag*_x_* I thin films are summarized in Figure 1. With increasing Ag^+^ incorporation (0–50.9 at.%), the films retain high transparency, as shown by the images in Figure 1a. The corresponding transmission spectra (Figure 1b) yield averaged visible‐light transmittance (400–800 nm) above 80% with a maximum over 90%. A slight red‐shift of the absorption edge from ∼410 nm for undoped CuI to ∼425 nm emerges for Ag^+^ content >8.8 at.%, indicating minor bandgap renormalization rather than degradation of optical transparency. Accordingly, the bandgap decreases from 3.01 eV for pristine CuI down to 2.81 eV for 50.9 at.% Ag‐doped Cu_1‐_ *_x_* Ag*_x_* I (Figure S1). Since all films have comparable thicknesses (136–153 nm; Figure S2), the optical evolution primarily arises from compositional effects. Field‐emission scanning electron microscopy (FE‐SEM) and energy‐dispersive spectroscopy (EDS) confirm a dense microstructure with uniform elemental distribution (Figure S2). Atomic force microscopy (AFM) (*x* = 0.088) reveals a smooth surface (RMS ∼11.0 nm; Figure S3), supporting transparent device integration and optical access through the device. X‐ray diffraction (XRD) (Figure 1c) shows all compositions retain the γ‐phase cubic CuI structure (PDF#06‐0246) with preferred (111) orientation. The lattice parameters are summarized in Figures S4–S6. Peak shifts to lower angles with increasing Ag reflect lattice expansion due to the larger Ag^+^ radius [31], without secondary phases up to *x* = 0.509 [32], which is expected to facilitate ionic transport.

**FIGURE 1:**
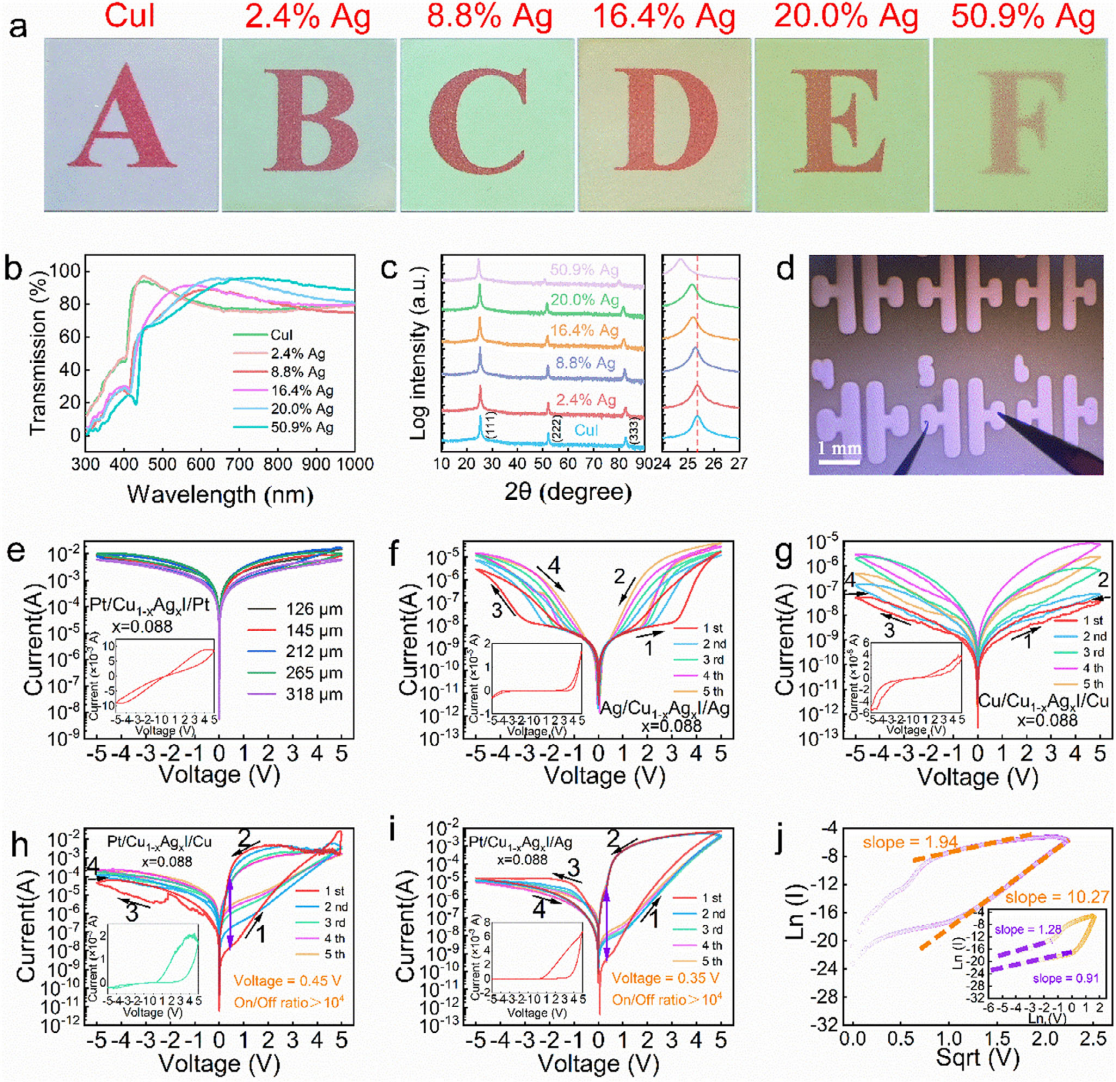
Structural, optical, and memristive properties of Cu_1‐_ *_x_* Ag*_x_* I thin films and devices. (a) Optical photographs of Cu_1‐_ *_x_* Ag*_x_* I thin films deposited on transparent glass substrates by room‐temperature magnetron sputtering. (b) Optical transmittance spectra (300–1000 nm) of films with different Ag doping concentrations. (c) X‐ray diffraction patterns of Cu_1‐_ *_x_* Ag*_x_* I thin films. (d) Photograph of the fabricated device together with a schematic illustration of the Cu_1‐_ *_x_* Ag*_x_* I‐based memristor structure on glass substrates. (e–g) *I*–*V* characteristics of symmetric Pt/Pt, Ag/Ag, and Cu/Cu devices based on Cu_1‐_ *_x_* Ag*_x_* I (*x* = 0.088) functional layers; symmetric Pt/Pt devices with different electrode spacings are used to evaluate geometric effects, while symmetric Ag/Ag and Cu/Cu devices are cycled for five consecutive sweeps to assess switching stability. (h, i) Resistive switching behavior of asymmetric Pt/Cu and Pt/Ag devices; corresponding linear‐scale *I*–*V* curves are shown in the insets. (j) $\sqrt{V}$‐*Ln I* characteristics of the Pt/Cu_1‐_ *_x_* Ag*_x_* I/Ag device under positive bias; the *Ln V*–*Ln I* relationship is shown in the inset.

To investigate how this compositionally engineered ionic transport influences resistive switching, planar memristive devices with in‐plane lateral electrode geometries were fabricated (Figure 1d). Unlike conventional vertical nanoscale architectures that favor localized CF formation, the present devices employ macroscopic electrode spacings (126–318 µm). This geometry increases the physical distance between the electrodes, strongly suppressing the likelihood of electrode‐spanning metallic bridging and providing a platform for probing distributed ionic redistribution and interfacial transport. Therefore, it is beneficial for studying the new mechanism by eliminating distractions from CF and provides a platform for probing distributed ion migration and interfacial transport processes in our designed transparent halide ionic semiconductors.

Electrical measurements were performed under voltage sweeps of 0 →5 → 0 → −5 → 0 V at room temperature. Representative current‐voltage characteristics for Pt/Cu_1‐_ *_x_* Ag*_x_* I/Pt devices are shown in Figure 1e (*x* = 0.088; Figure S7). Pristine CuI exhibits nearly linear *I*–*V* behavior with negligible hysteresis, consistent with quasi‐Ohmic Pt/CuI contacts. By contrast, Ag incorporation progressively introduces pronounced hysteresis together with increasing switching amplitude, reaching ∼10^2^ at *x* = 0.509. The absolute current decreases with increasing electrode spacing, consistent with the expected geometric scaling of distributed conduction (Figure S7), whereas no variation in the overall switching characteristics is observed within the investigated spacing range. Scaling down the electrode spacing would increase the lateral electric field at a given voltage and reduce the characteristic lateral length scale for ionic redistribution, thereby affecting the switching voltage and kinetics, while also reducing the device footprint and favoring higher‐density array integration. Hall measurements (Table S1) reveal that Ag incorporation continuously suppresses hole concentration from 3.78 × 10^19^ cm^−3^ in CuI to 1.57 × 10^17^ cm^−3^ at *x* = 0.509, while preserving p‐type transport. In contrast, the Hall mobility exhibits a non‐monotonic dependence on Ag content, increasing from 5.94 cm^2^ V^−1^ s^−1^ for pristine CuI to a maximum of 9.38 cm^2^ V^−1^ s^−1^ at *x* = 0.088, followed by a pronounced decrease at higher Ag concentrations. The initial mobility enhancement is consistent with the improved structural ordering at moderate Ag incorporation, whereas excessive Ag alloying introduces stronger lattice distortion and defect‐related carrier scattering. The enhanced mobility at *x* = 0.088 indicates favorable electronic transport in the composition that also exhibits the strongest resistive switching, although the switching behavior is not determined by carrier mobility alone. Ultraviolet photoelectron spectroscopy (Figures S8–S10) further shows that Ag incorporation modifies the interfacial energy‐level alignment through impurity‐state formation. These observations indicate that Ag incorporation simultaneously regulates bulk carrier density and interfacial energetics, thereby coupling ionic transport with electronic conduction. Correspondingly, Ag incorporation not only enhances ionic mobility through local lattice distortion and mixed‐cation transport, but also enables controllable modulation of the interfacial Schottky landscape responsible for analog switching.

To directly verify the role of this interfacial Schottky landscape, we replaced ohmic Pt with lower‐work‐function Ag or Cu top electrodes, thereby introducing rectifying Schottky contacts and back‐to‐back junction configurations in symmetric Ag/Cu_1‐_ *_x_* Ag*_x_* I/Ag and Cu/Cu_1‐_ *_x_* Ag*_x_* I/Cu devices [33, 34, 35, 36]. For *x* = 0.088 (Figure 1f,g), Ag devices show decreasing switching ratio with Ag content and eventual suppression at *x* = 0.509 (Figure S11), while Cu devices exhibit a non‐monotonic trend similar to Pt devices (Figure S12). Despite composition dependence, symmetric devices show limited contrast (∼10^2^) due to dual‐interface modulation. To break symmetry, asymmetric Pt/Cu_1‐_ *_x_* Ag*_x_* I/Cu and Pt/Cu_1‐_ *_x_* Ag*_x_* I/Ag structures were introduced, with bias applied to Pt while Cu/Ag is grounded. This differs from Electrochemical metallization (ECM) memories, where filaments nucleate from the active electrode under opposite polarity. Pt/Cu_1‐_ *_x_* Ag*_x_* I/Cu devices (*x* = 0.088) exhibit continuous conductance modulation over four orders of magnitude. Increasing Ag (x ≥ 0.20) weakens interfacial barriers (Figure S13), leading to more symmetric transport and cycling degradation due to ionic redistribution. Replacing Cu with Ag enhances asymmetry and suppresses reverse leakage, improving stability (Figure 1i; Figure S14). Pt/Cu_1‐_ *_x_* Ag*_x_* I/Ag devices (*x* = 0.088) show stable bipolar resistive states with ON/OFF ratios exceeding 10^4^ and 10^5^ are obtained when the devices are read at 0.35 and 0.58 V, respectively. At a fixed electrode spacing of 145 µm, increasing the electrode area from 0.06 to 0.22 mm^2^ increases the absolute current, while the overall switching characteristics remain largely unchanged (Figure S15). *I*–*V* analysis shows linear *Ln I*‐$V^{\frac{1}{2}}$ behavior at high bias (Figure 1j), while *Ln I*–*Ln V* gives slopes of 0.91 and 1.28 for HRS and LRS, indicating Schottky emission at high bias [37, 38], and Ohmic transport at low bias. Together with the gradual conductance modulation, these results support a mechanism dominated by distributed ionic redistribution and interfacial barrier modulation rather than localized filamentary conduction.

### 2.2 Field‐Driven Bulk Ionic Migration Governs Resistive Switching

Since the memristive characteristics establish device functionality, understanding the underlying ionic transport mechanism is essential for clarifying the origin of the analog switching behavior [39, 40, 41]. In conventional Cu‐based ECM memories, resistive switching is generally attributed to the formation and rupture of localized metallic CF [37, 42]. In contrast, the transparent Cu_1‐_ *_x_* Ag*_x_* I devices investigated here exhibit gradual conductance evolution and polarity inconsistent with classical filamentary switching, suggesting the involvement of a fundamentally different transport process.

To probe ionic redistribution, lateral elemental mapping was performed under applied bias. To enhance migration contrast, 8–10 V biases (above the normal operating range) were applied. As shown in Figure 2a, pristine devices exhibit homogeneous elemental distributions without detectable segregation (other elements in Figure S16). Under positive bias applied to the Pt electrode, Cu and Ag progressively accumulate near the grounded electrode, whereas iodine remains uniformly distributed throughout the channel (Figure 2b; Figure S17). A similar cation redistribution is observed in devices tested under opposite bias polarity (Figure 2c; Figure S18), suggesting a reversible tendency of cation redistribution under opposite bias polarity. A strong composition dependence is observed: pristine CuI exhibits predominantly Cu^+^ migration, while low Ag incorporation (*x* = 0.024 and 0.088) induces coupled Cu^+^/Ag^+^ transport. At higher Ag concentrations (*x* ≥ 0.164), ionic transport becomes increasingly Ag‐dominated (Figures S16–S19), indicating a crossover in the dominant mobile species. Temperature‐dependent electrochemical impedance spectroscopy (EIS) reveals a monotonic increase in ionic conductivity with Ag incorporation (Figure S20), consistent with enhanced superionic transport behavior [43], accompanied by a decrease in the transport activation energy from 0.51 eV for CuI to 0.30 eV at 42.2% Ag. Importantly, under reverse bias, with the Ag electrode positively biased and Pt grounded, metallic enrichment is observed at the grounded Pt interface, while no corresponding accumulation is detected at the positively biased Ag side (Figure S18). Notably, for CuI, only Cu is detected near the grounded Pt interface despite the Ag electrode being positively biased. For *x* = 0.024 and 0.088, both Cu and Ag are detected, whereas Ag becomes the dominant accumulated species at *x* ≥ 0.164. This composition‐dependent evolution of the redistributed species is consistent with the Cu_1‐_ *_x_* Ag*_x_* I functional‐layer composition and supports the functional layer as the primary source of the mobile cations. These observations support field‐driven ionic redistribution within the Cu_1‐_ *_x_* Ag*_x_* I layer. However, the switching performance does not increase monotonically with ionic conductivity, indicating that ionic transport alone does not determine the device response. At *x* = 0.088, the highest Hall mobility (9.38 cm^2^ V^−1^ s^−1^; Table S1) together with enhanced ionic transport favors effective conductance modulation, whereas further Ag incorporation markedly reduces carrier mobility and weakens the interfacial barrier. Thus, the optimal switching performance at *x* = 0.088 arises from a favorable balance between ionic transport and electronic/interfacial regulation.

**FIGURE 2:**
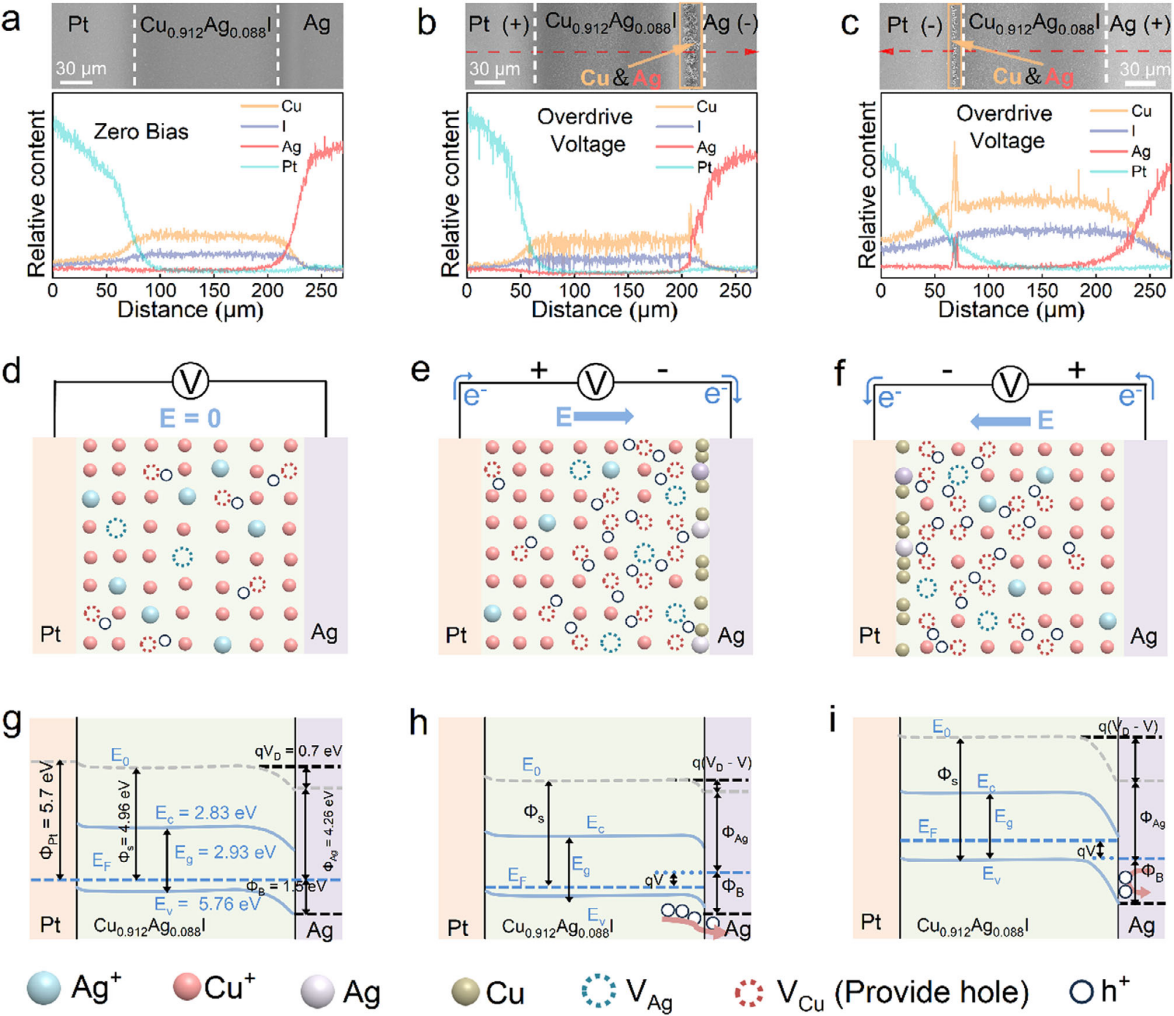
Ionic migration behavior and interfacial barrier modulation in Pt/Cu_1‐_ *_x_* Ag*_x_* I/Ag devices. (a–c) Lateral SEM images and corresponding EDS line profiles of Cu, I, Ag, and Pt in Pt/Cu_1‐_ *_x_* Ag*_x_* I/Ag (*x* = 0.088) devices: (a) pristine state, (b) after applying a positive bias, and (c) after applying a negative bias. (d–f) Schematic illustrations of electric‐field‐driven ion migration and redistribution processes within the Cu_1‐_ *_x_* Ag*_x_* I layer under different bias polarities. (g–i) Schematic energy band alignments of the Pt/Cu_1‐_ *_x_* Ag*_x_* I/Ag device at different steady‐state bias conditions.

The proposed switching mechanism is schematically illustrated in Figure 2d–f. Under forward bias, Cu^+^/Ag^+^ ions drift toward the cathodic interface and undergo interfacial reduction, leaving behind cation vacancies (V_Cu_ and V_Ag_) within the halide lattice. Because Cu_1‐_ *_x_* Ag*_x_* I is intrinsically p‐type, these vacancies act as acceptor‐like defects, increasing hole concentration and thereby lowering device resistance. Reversing the electric field drives ion back‐diffusion and partial vacancy refilling, restoring the high‐resistance state. Notably, no continuous metallic percolation pathways are observed throughout the switching process. Instead, the reduced species remain spatially confined near the grounded electrode, even after prolonged biasing (Figure 2b,c; Figures S17–S19). In conventional ECM switching, localized metallic nucleation can be followed by field‐enhanced, preferential ion migration and reduction at the growth front, leading to progressive filament extension toward the counter electrode [44, 45]. In contrast, the Cu/Ag accumulation observed here forms spatially confined, loose, and discontinuous deposits near the grounded interface rather than a continuous metallic pathway. This distinct accumulation behavior is not characteristic of the localized, self‐amplifying growth process associated with electrode‐bridging ECM filaments. Together with the gradual conductance evolution and the large lateral electrode spacing, these results support a filament‐free switching mechanism dominated by distributed ionic transport and electrochemical modulation of the bulk semiconductor.

The switching characteristics are further influenced by interfacial energetics. Owing to the high work function of Pt and the p‐type nature of Cu_1‐_ *_x_* Ag*_x_* I, Pt/Cu_1‐_ *_x_* Ag*_x_* I/Pt devices exhibit nearly Ohmic contacts, as supported by ultraviolet photoelectron spectroscopy. In these symmetric Pt devices, switching is governed primarily by bulk ionic redistribution and vacancy‐mediated carrier modulation. By contrast, Ag/Cu_1‐_ *_x_* Ag*_x_* I/Ag structures are dominated by Schottky barrier modulation because of the lower work function of Ag, leading to suppressed switching at high Ag contents. Cu/Cu_1‐_ *_x_* Ag*_x_* I/Cu devices exhibit intermediate behavior, evolving from barrier‐dominated transport at low Ag concentration toward increasingly bulk‐controlled conduction with further alloying. A markedly enhanced switching response emerges in asymmetric Pt/Cu_1‐_ *_x_* Ag*_x_* I/Ag architectures, where interfacial asymmetry cooperates with bulk ionic redistribution (Figure 2g–i). Pt serves as the electric‐field‐driving electrode, where forward bias facilitates hole injection and cation migration, whereas reverse bias suppresses carrier injection and partially restores the initial ionic configuration through the Schottky interface at Ag. This coupled modulation amplifies the conductance contrast while stabilizing analog switching behavior. Among all compositions, *x* = 0.088 provides the optimal balance between ionic mobility and interfacial barrier modulation, yielding switching ratios exceeding 10^4^ when read at 0.35 V and approaching 10^5^ when read at 0.58 V, together with excellent cycling stability. These results indicate that bipolar switching in Cu_1‐_ *_x_* Ag*_x_* I emerges from the interplay between distributed ionic transport and asymmetric interfacial electrochemistry.

To directly visualize ion dynamics under applied electrical bias, in situ TEM was performed on Cu_1‐_ *_x_* Ag*_x_* I (*x* = 0.088) devices using conductive Si substrates and Pt top electrodes. This geometry was used to directly visualize the field‐driven ionic response of the Cu_1‐_ *_x_* Ag*_x_* I functional layer, rather than to reproduce the full lateral device architecture. As shown in Figure 3a–c, electrically driven ion accumulation progressively develops near the interface under applied bias and continuously intensifies with the magnitude of the external electric field and over time. After removing the external field, partial relaxation of the accumulated species occurs owing to internal electrostatic restoring forces (Figure 3d,e), while residual redistributed ions remain trapped within the structure, consistent with the non‐volatile conductance modulation observed electrically. The full dynamic evolution is captured in Videos S1–S3. Under higher electric fields, pronounced interfacial ionic accumulation becomes clearly visible (Figure 3k–o). Corresponding EDS analysis confirms that the redistributed species are predominantly Cu, whereas iodine remains spatially uniform (Figure S21), supporting field‐driven Cu^+^ redistribution and interfacial reduction. HR‐TEM of the Cu‐rich regions identified by EDS further reveals well‐crystallized nanoparticles with clear lattice fringes. The corresponding FFT pattern shows lattice spacings of ∼0.208 and∼0.181 nm, corresponding to the (111) and (200) planes of face‐centered cubic Cu, respectively (Figure S22), supporting the reduction of migrated Cu^+^ to metallic Cu^0^. Importantly, a zero‐bias control experiment performed under the same electron‐beam energy, current density, and irradiation duration produces no obvious Cu/Ag redistribution or interfacial accumulation (Figure S23 and Video S4), indicating that the observed ionic redistribution is not induced by electron irradiation alone. Together, these in situ TEM observations directly visualize electrically driven cation redistribution and its partial relaxation after removal of the external field. Although the TEM geometry differs from that of the lateral Pt/Cu_1‐_ *_x_* Ag*_x_* I/Ag devices, the observed ionic dynamics are consistent with the spatial redistribution identified by EDS in the actual lateral devices. Thus, the in situ TEM provides direct visualization of the field‐driven ionic response of the functional layer, while the EDS measurements establish its occurrence in the lateral device architecture. Such spatially distributed ionic modulation enables continuous analog conductance evolution, which is advantageous for neuromorphic applications.

**FIGURE 3:**
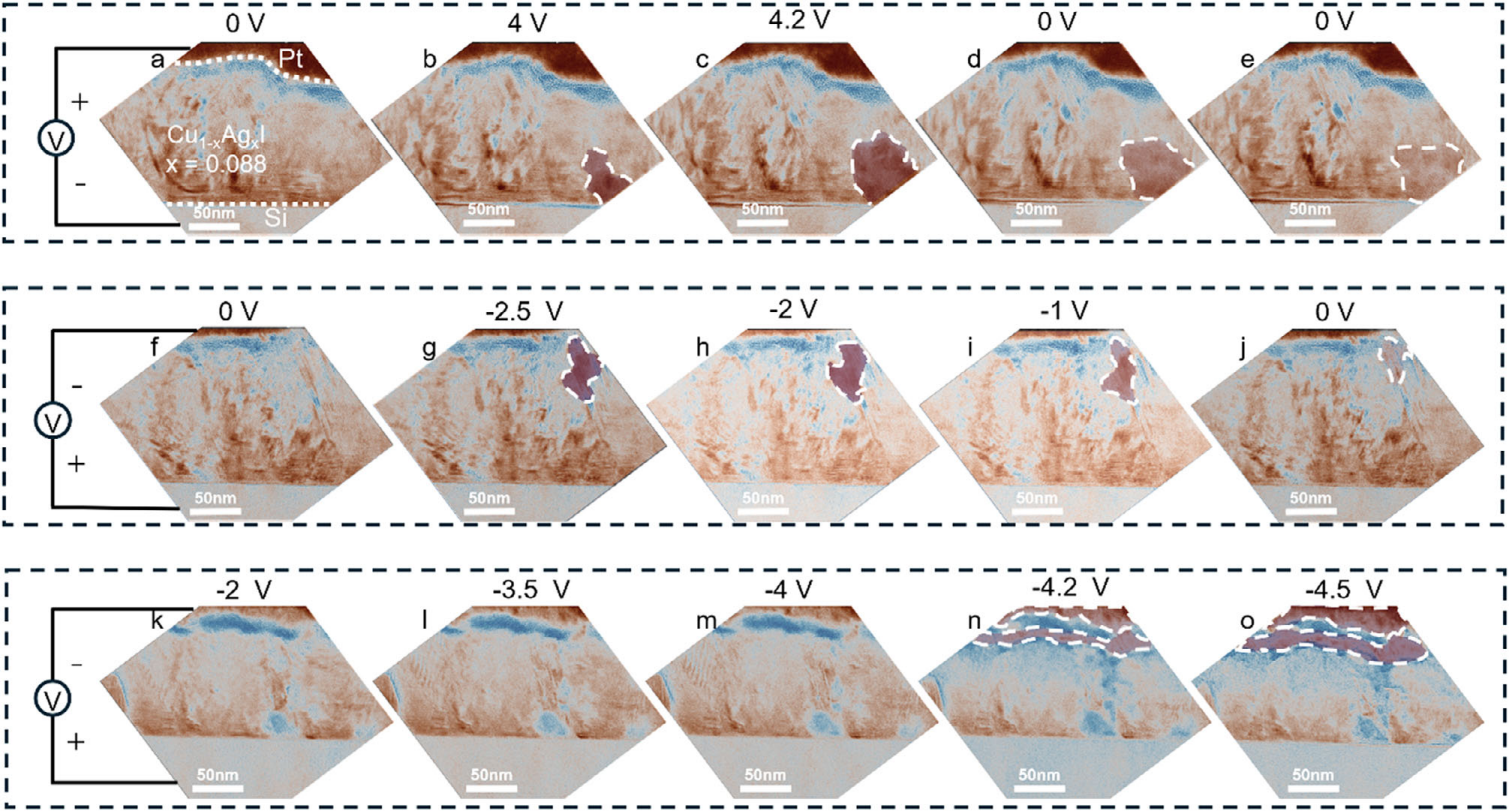
In situ transmission electron microscopy characterization of Cu_1‐_ *_x_* Ag*_x_* I (*x* = 0.088) thin films. (a–e) Sequential evolution of ionic migration and spatial redistribution under forward bias; (f–j) Dynamic ionic response and redistribution behavior under reverse bias; (k–o) Progressive ion accumulation and interfacial evolution under prolonged reverse bias stress.

### 2.3 Statistical Uniformity, Cycling Stability and Ion Migration Driven Switching

Despite rapid progress in memristive systems for neuromorphic and non‐von Neumann computing, stochastic CF formation remains a major obstacle because it introduces substantial device‐to‐device and cycle‐to‐cycle variability. Statistical analyses using an 8 × 10 device array (80 devices) and repeated switching measurements were therefore performed (Figure 4). At a read voltage of 0.35 V, the high‐resistance state (HRS) and low‐resistance state (LRS) are narrowly distributed around 4.8 × 10^8^and 3.5 × 10^4^ Ω, respectively, yielding a switching ratio of ∼2.7 × 10^4^ (Figure 4a–c). Repeated switching measurements further reveal stable bipolar characteristics over 80 consecutive cycles, with HRS and LRS values centered at 1.8 × 10^7^and 2.3 × 10^4^ Ω, respectively, corresponding to an average switching ratio of ∼8.1 × 10^2^ (Figure 4d–f). Long‐term retention measurements under ambient conditions show negligible degradation after 2 months, 1 year, and even 2 years, while maintaining switching ratios above 10^4^ throughout (Figures S24–S26). Such stability is inconsistent with stochastic metallic filament formation, instead supporting a spatially distributed ionic‐switching mechanism governed by reversible bulk ion migration and interfacial barrier reconstruction. To further clarify the ionic origin of resistive switching, the dependence of device behavior on voltage amplitude and sweep rate was investigated. As shown in Figure S27, enlarging the voltage window from ± 1 to ±5 V produces a pronounced enhancement of the *I*–*V* hysteresis, indicating progressively stronger field‐driven ionic redistribution within the Cu_1‐_ *_x_* Ag*_x_* I lattice. Sweep‐rate‐dependent measurements over a fixed voltage range of ±5 V (Figure S28) further show that reducing the sweep rate from 0.9 to 0.16 V·s^−1^ substantially increases conductance, demonstrating that slower electrical stimulation enables more complete ionic redistribution and more effective modulation of the interfacial electronic structure. Unlike abrupt filament nucleation in ECM memories, the conductance evolution remains smooth and continuous, consistent with the distributed ionic processes directly visualized by in situ TEM. Overall, the transparent Pt/Cu_1‐_ *_x_* Ag*_x_* I/Ag memristors exhibit exceptional uniformity, cycling stability, and ultralong retention, evidencing the intrinsic robustness of the filament‐free switching mechanism. Together with direct in situ TEM visualization of reversible ion migration, these findings demonstrate that resistive switching is governed by coupled bulk ionic redistribution and dynamically reconfigurable interfacial energy barriers, rather than stochastic CF formation characteristic of conventional CuI‐based ECM systems.

**FIGURE 4:**
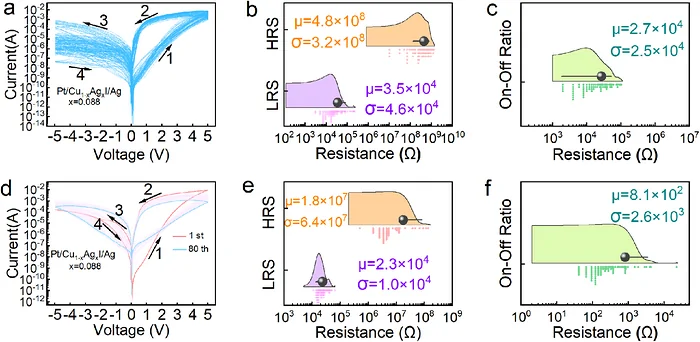
Uniformity and reproducibility characteristics of Pt/Cu_1‐_ *_x_* Ag*_x_* I/Ag memristive devices. (a) *I*–*V* characteristics from 80 individual Pt/Cu_1‐_ *_x_* Ag*_x_* I/Ag devices. (b, c) Device‐to‐device HRS and LRS distributions at 0.35 V and corresponding ON/OFF ratio. (d) Cyclic *I*–*V* curves of a representative Pt/Cu_1‐_ *_x_* Ag*_x_* I/Ag device over 80 cycles. (e, f) Statistical distributions of HRS, LRS at 0.35 V, and ON/OFF ratio.

### 2.4 Transparent Artificial Synapses Enabled by Ion‐Migration‐Induced Interfacial Barrier Modulation

The resistive switching of Pt/Cu_1‐_ *_x_* Ag*_x_* I/Ag memristors resembles synaptic weight modulation in biological systems, where neurotransmitter transport regulates synaptic transmission [46, 47]. In biological synapses, stimuli induce neurotransmitter diffusion across the synaptic cleft, modulating postsynaptic responses (Figure 5a). In Cu_1‐_ *_x_* Ag*_x_* I devices, electric‐field‐driven Cu^+^/Ag^+^ migration plays an analogous role by reconfiguring the interfacial energy landscape [48]. Unlike filamentary memristors, the synaptic response originates from reversible ionic redistribution and interfacial barrier modulation, consistent with in situ TEM observations. For *x* = 0.088, device synaptic properties were evaluated. At 0.35 V read bias, both HRS and LRS remain stable for > 4 × 10^4^ s with a switching ratio ∼10^2^ (Figure 5b; Figure S29), reflecting stable interfacial barrier reconstruction without stochastic filament formation. Short‐term plasticity was examined via paired‐pulse facilitation (PPF) [46, 49, 50]. As shown in Figure 5c, the PPF index decreases exponentially with interpulse interval ΔT (1–100 ms) under 5 V, 100 ms programming pulses and follows a double‐exponential model:

(1) $$PPF\;index=y_{0}+C_{1}e^{\frac{-\Delta T}{\tau_{1}}}+C2e^{\frac{-\Delta T}{\tau_{2}}}$$

where C_1_ and C_2_ are facilitation constants, y_0_ is the baseline, and τ_1_ and τ_2_ represent fast and slow relaxation processes [51, 52]. Extracted values (τ_1_ = 7.54 ms and τ_2_ = 60.83 ms) indicate multiscale ionic relaxation associated with local redistribution and interfacial equilibration [46, 53]. Long‐term synaptic plasticity was demonstrated through pulse‐train measurements. Successive positive programming pulses (+ 5 V, 100 ms) progressively increase the postsynaptic current (PSC), corresponding to long‐term potentiation (LTP), whereas negative programming pulses gradually reverse the conductance state and induce long‐term depression (LTD) (Figure 5d). Quantitative fitting of the LTP/LTD characteristics gives nonlinearity factors of α_LTP_ ≈ ‐3.10 and α_LTD_ ≈ 7.98, corresponding to an asymmetry factor of 4.88 between the potentiation and depression processes (Figure S30). The corresponding cycle‐to‐cycle (C2C) and device‐to‐device (D2D) variations are further quantified in Figure S31. Using a 3σ criterion based on the D2D conductance variation [54], eight and five statistically distinguishable conductance levels are obtained for potentiation and depression, respectively. The programming energy is calculated to be 14.1 nJ per pulse based on the minimum programming current in Figure 5d. The performance of the device is benchmarked against representative memristive devices in Table S3. During repeated stimulation, relaxation time increases (Figure 5e), suggesting gradual stabilization of ionic configurations. Pulse‐dependent measurements show that larger stimuli yield stronger conductance modulation with saturation behavior (Figure 5f), analogous to synaptic habituation [21]. Conversely, weaker stimulation results in faster conductance decay after negative pulses. Additional analyses (Figures S32 and S33) confirm adaptive and continuously tunable conductance states governed by ionic transport kinetics and interfacial barrier evolution, thereby providing a multidimensional basis for analog synaptic weight regulation and neuromorphic computation [52]. Sensitization behavior is observed: repeated stimulation enhances conductance with diminishing gain, while stronger pulses trigger abrupt increases (Figure 5g), attributed to field‐assisted reconstruction of migration pathways and interfacial barriers [55, 56, 57]. Despite the measurable nonlinearity and asymmetry of the weight updates, the conductance can be continuously tuned in both directions with reproducible modulation, consistent with distributed ionic dynamics. To evaluate neuromorphic performance, a crossbar neural network based on Pt/Cu_1‐_ *_x_* Ag*_x_* I/Ag synapses (*x* = 0.088) was implemented for the MNIST dataset (Figure 5h), with a 784‐100‐10 architecture (Figure S34). After 100 epochs, the network achieves 98% accuracy (Figure 5i), confirming reliable analog weight updates enabled by reversible ionic redistribution and interfacial modulation. Beyond the demonstrated neuromorphic functionality, the high visible transmittance of the Cu_1‐_ *_x_* Ag*_x_* I devices provides optical access through the memory layer, offering a route to neuromorphic elements that can be integrated with transparent sensors, displays, or imaging interfaces while minimizing visual obstruction. Overall, these results establish transparent Cu_1‐_ *_x_* Ag*_x_* I synapses operating via non‐filamentary ion migration coupled with reconfigurable interfacial barriers, providing a materials and device platform for optically compatible neuromorphic systems.

**FIGURE 5:**
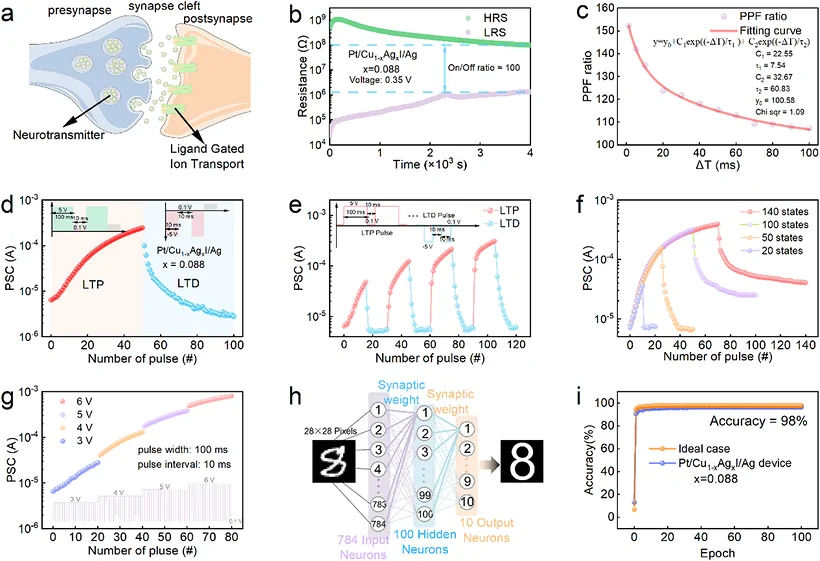
Neuromorphic characteristics of the Pt/Cu_1‐_ *_x_* Ag*_x_* I/Ag (*x* = 0.088) synaptic device. (a) Schematic illustration of a biological neural synapse. (b) Long‐term retention characteristics of the artificial synapse. (c) Paired‐pulse facilitation (PPF) response. (d) LTP and LTD programmed by a train of positive (+5 V, 100 ms) and negative (−5 V, 10 ms) voltage pulses, respectively; a 0.1 V read voltage is applied between programming pulses. (e) Cycling endurance of the LTP/LTD characteristics. (f) Programmable LTP and LTD behaviors under pulse trains with varying numbers. (g) Emulation of biological habituation and sensitization. (h) A schematic diagram of a three‐layer artificial neural network. (i) Performance of an artificial neural network constructed using the device for recognizing 8 × 8 pixel handwritten digits, showing accuracy evolution over training epochs.

## 3 Conclusion

In summary, we address key materials and device challenges associated with p‐type transparent neuromorphic systems by introducing an alloyed Cu_1‐_ *_x_* Ag*_x_* I s solid‐state ionic platform. By leveraging Ag^+^ compositional engineering, we simultaneously tuned the carrier density and electronic structure of CuI while preserving its γ‐phase, effectively activating room‐temperature ionic transport that is otherwise latent in the undoped semiconductor. This material design transforms CuI from a predominantly hole‐conducting transparent semiconductor into an ionically active synaptic platform at room temperature. Crucially, in situ TEM and EDS mapping provided atomic‐scale visualization of homogeneous Cu^+^/Ag^+^ redistribution, confirming that resistive switching is governed by bulk ion migration coupled with interfacial energy barriers, rather than stochastic filament formation. The optimized asymmetric Pt/Cu_1‐_ *_x_* Ag*_x_* I/Ag devices (*x* = 0.088) exhibit a switching ratio exceeding 10^5^, exceptional retention beyond two years, and reliable emulation of synaptic plasticity. More broadly, this work establishes a materials design strategy for p‐type transparent memristors, wherein rational alloying of halide ionic semiconductors enables distributed ionic transport for reliable analog switching and provides a potential route toward optically compatible neuromorphic systems.

## 4 Methods

### 4.1 Fabrication of Cu_1‐_ *_x_* Ag*_x_* I Thin Films and Devices

Cu_1‐_ *_x_* Ag*_x_* I thin films and memristive devices were fabricated using a custom‐designed glovebox‐integrated magnetron sputtering system. Sputtering targets with various Ag compositions were prepared from CuI (AR ≥ 99.5%, Aladdin) and AgI (AR ≥ 99%, Macklin) precursor powders. During thin‐film deposition, the base pressure of the chamber was maintained at 3 × 10^−4^ Pa. The films were deposited onto transparent glass substrates at room temperature, which had been sequentially cleaned with deionized water, acetone, isopropanol, and anhydrous ethanol. The sputtering pressure was 1 Pa, and the RF power was 20 W, yielding compact and uniform Cu_1‐x_Ag_x_I films with controlled thickness. Metal electrodes (Pt, Cu, and Ag; purity > 99%, Prmat (Shanghai) Technology Co., Ltd.) were subsequently deposited through a shadow mask using DC magnetron sputtering at room temperature, 1 Pa, and 70 W. The resulting device architecture is illustrated in Figure 1d. The device geometry is illustrated in Figure 1d. The metal electrodes have a strip‐like geometry with lateral probe‐contact extensions, a length of 1200 µm, a width of 200 µm, and a thickness of approximately 120 nm. The lateral electrode spacing was varied as specified in the corresponding measurements.

### 4.2 Structural and Surface Characterization

XRD patterns were recorded using a PANalytical Empyrean diffractometer (Netherlands) equipped with a Cu Kα radiation source. Surface and cross‐sectional morphologies were examined using field‐emission scanning electron microscopy (FE‐SEM, Zeiss GeminiSEM 450, Germany).

Surface roughness was evaluated using atomic force microscopy (AFM, Park NX10, Korea). After ∼5 nm surface etching, ultraviolet photoelectron spectroscopy (UPS, Thermo Fisher ESCALAB XI^+^, USA) was employed to analyze the work function and valence‐band spectra. Optical transmittance and bandgap information were obtained using a UV–vis spectrophotometer (Hitachi U‐3900, Japan).

### 4.3 In Situ Transmission Electron Microscopy Characterization

Specimens were prepared by focused ion beam milling using a dual‐beam system (FEI Helios G4). Ion migration under an electric field was examined by aberration‐corrected TEM (ARM‐300F, JEOL), while elemental distributions after electrical overstress were analyzed using (JEM‐2100F, JEOL).

### 4.4 Electrical Measurements

*I*–*V* measurements and pulse‐dependent synaptic tests were performed at room temperature using a Keysight 4200A‐SCS (Keithley, USA) semiconductor parameter analyzer, in conjunction with a Lake Shore TTPX probe station. Resistive‐switching measurements were conducted using a voltage sweep sequence of 0 →5 → 0 → ‐5 → 0 V with a voltage step of 0.005 V and a compliance current of 0.1 A. Intrinsic electrical properties of the films were measured using a Linseis HCS1 system (Germany).

EIS for ion conductivity extraction was conducted at room temperature using a Metrohm Multi AUTOLAB M204 workstation (Switzerland), and impedance spectra were fitted using ZSimpWin.

For D2D statistical analysis, 20 devices were selected from each of four independently fabricated batches, giving a total of 80 devices.

## Author Contributions

**Yuzhao Ouyang**: data curation, Writing – original draft, visualization, methodology, validation, conceptualization, investigation, software. **Ke Qu**: methodology, data curation, formal analysis, writing – original draft. **Haonan Wang**: software, visualization, data curation, writing – original draft. **Jianquan Liu**: methodology, software. **Wei Li**: data curation, software. **Zhuoran Ji**: investigation. **Jialin Yang**: investigation, formal analysis. **Qiuxiang Zhu**: conceptualization, investigation, supervision. **Zhenzhong Yang**: supervision, methodology, data curation, writing – review and editing, funding acquisition. **Xiaodong Tang**: conceptualization, formal analysis. **Hui Peng**: writing – review and editing. **Bobo Tian**: funding acquisition, resources, visualization, conceptualization. **Chungang Duan**: writing – review and editing, funding acquisition, resources, project administration, investigation. **Chang Yang**: project administration, resources, funding acquisition, writing – review and editing, validation, supervision, investigation, formal analysis, methodology, conceptualization.

## Conflicts of Interest

The authors declare no conflicts of interest.

## Supporting information




**Supporting File 1**: advs78209‐sup‐0001‐SuppMat.docx.

**Supporting File 2**: advs78209‐sup‐0002‐VideoS1.mp4.

**Supporting File 3**: advs78209‐sup‐0003‐VideoS2.mp4.

**Supporting File 4**: advs78209‐sup‐0004‐VideoS3.mp4.

**Supporting File 5**: advs78209‐sup‐0005‐VideoS4.mp4.

## Data Availability

The data that support the findings of this study are available from the corresponding author upon reasonable request.
