# Supplementary material for: Lattice Disorder in CuI Enables Filament‐Free Transparent Memristors
Source: Adv Sci (Weinh). 2026 Oct 11:e78209. Online ahead of print. doi: 10.1002/advs.78209 (PMC13655244; doi:10.1002/advs.78209)
Supplement: Supplementary file 1 [file ADVS-9999-e78209-s003.docx]

**Supporting Information**

**Lattice Disorder in CuI Enables Filament-free Transparent Memristors**

Yuzhao Ouyang^a,b#^, Ke Qu^a#^, Haonan Wang^a#^, Jianquan Liu^a.b^, Wei Li ^a.b^, Zhuoran Ji^a^, Jialin Yang^a,c^, Qiuxiang Zhu^a,b^, Zhenzhong Yang^a^, Xiaodong Tang^a,d^, Hui Peng^a,b,d^, Bobo Tian^a,b^*, Chungang Duan^a,b,d,^*, Chang Yang^a,b,^*

*^a^Key Laboratory of Polar Materials and Devices (MOE), Department of Electronics, School of Information and Electronic Engineering, East China Normal University, Shanghai, 200241, China.*

*^b^Shanghai Centre of Brain-inspired Intelligent Materials and Devices, East China Normal University, Shanghai, 200241, China.*

*^c^Shanghai Transparent Semiconductor Technology Co., Ltd., Building 1, No. 58 Yuanmei Road, Minhang District, Shanghai 200241, China.*

*^d^Collaborative Innovation Center of Extreme Optics, Shanxi University, Taiyuan, Shanxi 030006, China.*

#These authors contributed equally to this work.

* Corresponding author.

Email: cyang@phy.ecnu.edu.cn (C.Y.), cgduan@clpm.ecnu.edu.cn (C.D.), bbtian@ee.ecnu.edu.cn (B.T,)

**Figure S1. Optical bandgap analysis of Cu_1-_*_x_*Ag*_x_*I thin films.** (a) Tauc-Plot fitting curves for optical bandgap determination; (b) Variation of the optical bandgap (Eg) of Cu_1-_*_x_*Ag*_x_*I thin films as a function of Ag doping content (*x*).

To systematically investigate the bandgap modulation mechanism in Cu_1-_*_x_*Ag*_x_*I thin films induced by Ag⁺ doping, we employed the Tauc-Raju formulation (S1) for optical bandgap determination: ^[1, 2]^:

${(\alpha h\nu)}^{\frac{1}{n}}$ = A (hν - E_g_)	(S1)

where h denotes Planck's constant, α represents the absorption coefficient, ν is the photon frequency, A stands for a material-specific constant, and E_g_ corresponds to the optical bandgap. Distinct fitting procedures were implemented for direct (n=1/2) and indirect (n=2) bandgap semiconductors. Through meticulous Tauc plot extrapolation (Fig. S1(b)), we precisely determined the optical bandgaps across varying Ag⁺ concentrations (Fig. 1S (c)), revealing a consistent bandgap narrowing from ~3.01 eV to ~2.81 eV with increasing Ag⁺ content (0 → 50.9 at.%). This bandgap reduction phenomenon originates primarily from the formation of impurity energy states within the forbidden gap through Ag⁺ incorporation, effectively lowering the apparent optical bandgap. Notably, as AgI is a characteristic n-type semiconductor where electronic conduction arises from activated electrons of Ag⁺ Frenkel defects, ^[3, 4]^ the Ag⁺ doping simultaneously induces a substantial decrease in carrier concentration (as verified by Hall effect measurements, Supporting Information Table S1). This dual modulation of both band structure and charge transport properties provides a crucial foundation for: (i) the rational design of memristive devices based on Cu_1-_*_x_*Ag*_x_*I systems, and (ii) the precise electric-field control of memristive switching characteristics through carrier concentration engineering.

**Figure S2. SEM surface morphology and EDS scanning spectrum results of CuI thin films with different Ag doping concentrations.** (a, g) pristine CuI, (b, h) 2.4% Ag, (c, i) 8.8% Ag, (d, j) 16.4% Ag, (e, k) 20.0% Ag, and (f, l) 50.9% Ag. (m-r) show the corresponding cross-sectional SEM images.

Fig S2 presents the surface morphology and elemental distribution characteristics of Cu_1-_*_x_*Ag*_x_*I thin films with varying Ag doping ratios (*x*=0-0.509). All films prepared by room-temperature magnetron sputtering exhibit excellent densification and uniform grain size distribution. Notably, undoped CuI films display characteristic triangular grains due to the lowest surface energy of (111) crystallographic planes, while this distinctive morphology disappears upon Ag incorporation. XRD analysis (The data is sourced from Fig 1b) combined with Scherrer equation calculations (Fig S2) reveals a decreasing trend in crystallite size with increasing Ag content. However, SEM observations show an apparent increase in grain size with Ag doping, a seeming contradiction that may originate from sub-resolution polycrystalline aggregates in heavily Ag-doped Cu_1-_*_x_*Ag*_x_*I films. Atomic force microscopy (AFM) characterization (Fig S3) corroborates the XRD results, demonstrating a genuine reduction in grain size with Ag incorporation and confirming the exclusive presence of isolated triangular grains in pure CuI films. EDS quantitative analysis (Fig S2) confirms homogeneous elemental (Cu, Ag, I) distribution without segregation, with measured Ag/(Cu+Ag) atomic ratios of 0 %, 2.4 at.%, 8.8 at.%, 16.4 at.%, 20.0 at.%, and 50.9 at.%, showing excellent agreement with designed doping concentrations. Cross-sectional SEM images (Figs f-j) indicate film thickness variations within 136-153 nm, ensuring negligible impact on optical transmittance and device performance. All films exhibit sharp interfaces with glass substrates, demonstrating superior interfacial adhesion.

**Figure S3. AFM surface topography of Cu_1-_*_x_*Ag*_x_*I thin films.** (a) pristine CuI, (b–f) Ag-doped Cu_1-_*_x_*Ag*_x_*I with Ag^+^ concentrations of 2.4%, 8.8%, 16.4%, 20.0%, and 50.9%, respectively.

As shown in Fig S3, the vertical morphology of Cu_1-_*_x_*Ag*_x_*I thin films was characterized by AFM with a scan area of 10 × 10 μm. The root-mean-square roughness (Rq) values were measured to be 10.0 nm (CuI), 4.8 nm (2.4% Ag), 11.0 nm (8.8% Ag), 11.5 nm (16.4% Ag), 15.0 nm (20.0% Ag), and 12.2 nm (50.9% Ag). The results indicate that moderate Ag⁺ doping effectively reduces surface roughness, whereas excessive Ag⁺ incorporation leads to its resurgence. This trend may be attributed to Ag⁺ induced grain refinement: higher Ag⁺ concentrations decrease grain size and weaken intergranular connectivity, thereby promoting surface irregularity.

**Figure S4. The lattice parameters of Cu_1-_*_x_*Ag*_x_*I thin films grown on glass substrates at different Ag doping levels.** (a) undoped CuI, (b) 2.4 at%, (c) 8.8 at%, (d) 16.4 at%, (e) 20.0 at%, and (f) 50.9 at%.

In the 2θ-ω scans, all components exhibit characteristic cubic crystal structure. The out-of-plane lattice parameter of Cu_1-_*_x_*Ag*_x_*I thin films can be determined using the cubic crystal formula (S2):

$a=d_{hklA}\sqrt{h^{2}+k^{2}+l^{2}}$	(S2)

where a represents the lattice parameter, d_hkl_ denotes the interplanar spacing of (hkl) planes, and (hkl) are the corresponding Miller indices.

To minimize systematic errors from the measurement instrument, an extrapolation function (S3) was employed for linear fitting of the lattice parameters calculated from each diffraction peak: ^[5]^

$a= f({cos}^{2}\theta)$	(S3)

The instrument-error-corrected lattice parameters were obtained by linearly extrapolating to cos²θ = 0 (i.e., θ = 90°), with the corrected data presented in Fig S5.

**Figure S5. Revised lattice parameters of distinct compositional phases.**

The lattice parameters of Cu_1-_*_x_*Ag*_x_*I thin films exhibit a pronounced increasing trend with rising Ag⁺ doping concentration, expanding from 6.052 Å for the undoped sample to 6.226 Å at 50.9% Ag content. This lattice expansion can be attributed to the larger ionic radius of Ag⁺ (1.45 Å) compared to Cu⁺ (1.32 Å).

**Figure S6. Full width at half maximum and lattice size of different Ag contents in thin films.**

The research indicates that the crystallinity of the thin films deteriorates with increasing Ag doping concentration, accompanied by a notable reduction in grain size. However, when the Ag doping concentration is optimized (e.g., 8.8 at.%), the crystalline quality of the films can be significantly enhanced.

**Figure S7. Resistive switching characteristics of Pt-Pt symmetric electrode devices with different electrode spacings measured at room temperature.** (a) CuI, (b) 2.4% Ag, (c) 16.4% Ag, (d) 20.0% Ag, and (e) 50.9% Ag functional layers. (f) Comparison of the resistive switching behavior for all compositions at an electrode spacing of 145 μm (the data for 8.8% Ag are taken from Fig 2a in the main text).

The resistive switching characteristics of Pt/Cu_1-_*_x_*Ag*_x_*I/Pt devices with different functional layers and electrode spacings are presented in Fig S7. For a given functional layer, increasing the electrode spacing decreases the absolute current, consistent with the expected geometric dependence of a distributed conducting channel. Within the investigated spacing range, however, the overall hysteretic switching behavior shows no pronounced systematic dependence on electrode spacing. When devices with the same electrode spacing (145 μm) are compared across compositions (Fig. S7f), the switching hysteresis and switching ratio increase progressively with Ag content.

**Figure S8. Optical characterization of the AgI thin film.** (a) Optical transmittance spectrum of the AgI thin film in the UV-visible region. (b) Tauc plot derived from the absorption spectrum for extraction of the optical bandgap.

To elucidate the evolution of the work function and band structure of Cu_1-_*_x_*Ag*_x_*I thin films, ultraviolet-visible absorption (UV-Vis) measurements were performed. According to Equation S1, the optical bandgap of the AgI film was estimated to be approximately 2.77 eV, and the optical bandgaps of the other compositions are shown in Fig S1.

**Figure S9. Room-temperature ultraviolet photoelectron spectroscopy (UPS) of Cu_1-_*_x_*Ag*_x_*I (*x* = 0, 0.088, 0.20, and 1).** (a) Secondary electron cutoff region (the inset shows the corresponding full spectra). (b) Valence band spectra in the vicinity of the Fermi level.

The valence band maximum (VBM) and Fermi level positions were obtained from ultraviolet photoelectron spectroscopy (UPS) spectra, calibrated with respect to the He I photon energy (21.2 eV), as shown in Figs S9a and S9b. Based on the definitions of the high-binding-energy cutoff and the low-binding-energy onset, the VBM values relative to the vacuum level for Cu_1-_*_x_*Ag*_x_*I films (*x* = 0, 0.088, 0.20, and 1) were determined to be 6.10 eV, 5.76 eV, 5.63 eV, and 6.02 eV, respectively. The corresponding Fermi levels were 0.74 eV, 0.80 eV, 0.96 eV, and 1.98 eV. These results demonstrate a systematic evolution of the electronic structure with Ag incorporation, reflecting the modulation of the valence band and work function in Cu_1-_*_x_*Ag*_x_*I films.

**Figure S10. Schematic band structures of Cu_1-_*_x_*Ag*_x_*I thin films (*x* = 0, 0.088, 0.20, and 1).**

Based on the data obtained from Figs S1, S8, and S10, the schematic energy band diagrams of Cu_1-_*_x_*Ag*_x_*I thin films (*x* = 0, 0.088, 0.20, and 1) were constructed, as shown in Fig S10. The work function gradually decreases from 5.36 eV to 4.04 eV with increasing Ag content. According to the Hall measurement results (Table S1), when the Ag content in the films is ≤ 50.9%, both the Hall mobility and carrier concentration are positive, confirming that all samples retain p-type (hole) conduction behavior. Considering that the work function of Pt is 5.68 eV, it can be inferred that, in the absence of Fermi-level pinning and chemical disorder, Pt forms an Ohmic contact with Cu_1-_*_x_*Ag*_x_*I films (*x* = 0, 0.024, 0.164, 0.20, and 0.509). It is noteworthy that, as shown in Fig S10d, the AgI film exhibits n-type (electron) conduction, indicating that with increasing *x*, the dominant carrier type in Cu_1-_*_x_*Ag*_x_*I gradually transitions from holes to electrons. This transition is likely accompanied by structural changes in the crystal lattice, and the detailed correlation between carrier type and crystal structure will be discussed in our future work.

**Figure S11. Resistive switching characteristics of Ag/Cu_1-_*_x_*Ag*_x_*I/Ag (*x* = 0, 0.024, 0.164, 0.20, and 0.509) memristive devices.**

In Ag/Cu_1-_*_x_*Ag*_x_*I/Ag (*x* = 0, 0.024, 0.164, 0.20, and 0.509) devices, an interfacial barrier forms between the Ag electrodes and the Cu_1-_*_x_*Ag*_x_*I functional layer, and the resistive switching behavior is predominantly governed by modulation of this barrier. During the initial *I-V* cycle, Ag1 and Ag2 are designated as the positive and negative electrodes, respectively. In process 1 (0 → 5 V), the continuous voltage applied at Ag1 drives Cu⁺ or Ag⁺ ions toward Ag2. This ion migration induces an n-type doping-like effect near Ag2, thereby reducing its work function. Simultaneously, the ions that reach Ag2 are reduced to metallic Cu or Ag, further modifying the Ag2/functional-layer interfacial barrier. Consequently, the interfacial barrier height decreases and the device exhibits a nonlinear *I-V* characteristic. In process 2 (5 V → 0), the interface remains in a low-barrier state, enabling continued ion migration from Ag1 to Ag2. The resulting increase in current relative to process 1 leads to the SET transition. In process 3 (0 → -5 V), the migration direction reverses. At the onset of the negative sweep, the device retains the low-resistance state produced in process 2, allowing a momentarily high current. As ions gradually return to their original sites, the resistance of the functional layer increases, while the magnitude of the applied negative voltage continues to rise. This combination results in a slow increase in current with increasing negative bias. Under this reversed bias, a large number of Cu or Ag vacancies accumulate near Ag1, enabling rapid refilling by mobile ions and, in some cases, the re-reduction of metal ions previously migrated from Ag2. In process 4 (-5 V → 0), because the initial restoration of ion positions has been largely completed during process 3 and ion migration is reversible, ions continue to move toward Ag1, further reducing the resistance of the functional layer and producing another SET event. After the first I–V cycle, ions accumulate near Ag1, yielding a resistance lower than the pristine state. Consequently, in subsequent cycles, the overall current becomes higher than that observed in the first sweep. It is worth noting that, due to the symmetric interfacial barriers, the device exhibits highly symmetric *I-V* characteristics under positive and negative bias. Moreover, the current in Ag/Cu_1-_*_x_*Ag*_x_*I/Ag devices is significantly lower than that in Pt/Cu_1-_*_x_*Ag*_x_*I/Pt devices.

**Figure S12. Resistive switching characteristics of Cu/Cu_1-_*_x_*Ag*_x_*I/Cu (*x* = 0, 0.024, 0.164, 0.20, and 0.509) memristive devices.**

As presented in Fig. S12, all Cu_1-_*_x_*Ag*_x_*I compositions exhibit highly symmetric *I-V* characteristics around 0 V, owing to the symmetric Cu-Cu parallel electrode configuration. After five consecutive voltage sweeps, the overall conductance of the devices increases markedly, indicating sustained activation of ion migration. Furthermore, all compositions maintain an ON/OFF ratio of approximately 10^2^. Despite this reasonably high switching ratio, the Cu/Cu_1-_*_x_*Ag*_x_*I/Cu devices exhibit pronounced volatility, which compromises their data retention capability. Similar volatile behavior is also observed in Pt/Cu_1-_*_x_*Ag*_x_*I/Pt and Ag/Cu_1-_*_x_*Ag*_x_*I/Ag devices.

**Figure S13. Resistive switching characteristics of Pt/Cu_1-_*_x_*Ag*_x_*I/Cu (*x* = 0, 0.024, 0.164, 0.20, and 0.509) memristive devices.**

Based on the interfacial barrier characteristics between the functional layer and the metal electrodes, asymmetric Pt/Cu_1-_*_x_*Ag*_x_*I/Cu synaptic devices were constructed. When x ≤ 0.164, the work function of the Cu_1-_*_x_*Ag*_x_*I functional layer exceeds that of Cu, resulting in a pronounced interfacial barrier. As illustrated in Fig. S13(a-c), the *I-V* cycling curves exhibit a clear rectifying behavior: the current in the negative voltage region is approximately two orders of magnitude lower than that in the positive region, which primarily arises from the Cu/Cu_1-_*_x_*Ag*_x_*I interfacial barrier. At *x* = 0.20, the work function of Cu_1-_*_x_*Ag*_x_*I becomes comparable to that of Cu and continues to decrease with increasing Ag content. Consequently, when x ≥ 0.20, no significant interfacial barrier is expected at the metal/semiconductor interface. The corresponding *I-V* behavior, shown in Fig. S13(d-e), displays the anticipated symmetry under positive and negative bias, while the observed switching ratio is attributed to the migration of Cu or Ag ions. It is noteworthy that all Pt/Cu_1-_*_x_*Ag*_x_*I/Cu devices demonstrate pronounced volatility during *I-V* measurements. We speculate that this behavior may originate from the relatively high work function of Cu, which weakens its ability to effectively suppress current injection and ion migration under negative bias.

**Figure S14. Resistive switching characteristics of Pt/Cu_1-_*_x_*Ag*_x_*I/Ag (x = 0, 0.024, 0.164, 0.20, and 0.509) memristive devices.**

To improve the stability and enhance the switching ratio of Cu_1-_*_x_*Ag*_x_*I when employed as the functional layer in artificial synapses, we adopted a Pt/Cu_1-_*_x_*Ag*_x_*I/Ag device architecture. The relatively low work function of the Ag electrode (4.26 eV) induces a strong interfacial asymmetry, giving rise to pronounced rectification in all devices. As shown in Fig. S14a-c, devices with x ≤ 0.164 exhibit well defined nonvolatile switching. This behavior stems from the fact that, under negative bias, the majority of the potential drop localizes at the Ag/Cu_1-_*_x_*Ag*_x_*I interface, which effectively suppresses the rapid backward migration of cations and thus stabilizes the conductive state. Under a read voltage of 0.35 V, these devices deliver an ON/OFF ratio exceeding 10^4^. During repeated cycling, ion activation induced by five consecutive *I-V* sweeps results in a noticeable increase in the high resistance state (HRS) conductance, whereas the low resistance state (LRS) remains largely unaffected. When x ≤ 0.20, the substantial reduction in carrier concentration lowers the LRS conductance and suppresses the switching ratio, and the devices transition to volatile behavior during repeated sweeps. These results identify the ionic conductivity of the functional layer and the interfacial barrier height as the primary factors governing switching stability and nonvolatility in Cu_1-_*_x_*Ag*_x_*I-based devices. A comprehensive evaluation of switching ratio, cycling endurance, and retention characteristics indicates that the Pt/Cu_1-_*_x_*Ag*_x_*I/Ag device with *x* = 0.088 delivers the most balanced and robust performance. Consequently, this composition was selected for the subsequent neuromorphic synaptic emulation.

**Figure S15. Resistive switching characteristics of Pt-Ag electrode devices with different electrode-area measured at room temperature.** (a) CuI, (b) 2.4% Ag, (c) 16.4% Ag, (d) 20.0% Ag, and (e) 50.9% Ag functional layers.

To further examine the geometric dependence of conduction and switching, devices with different electrode areas of 0.06, 0.12 and 0.22 mm^2^ were characterized at a fixed electrode spacing of 145 μm. Across all investigated Ag compositions, the absolute current systematically increases with electrode area. The 0.22 mm^2^ devices exhibit the highest current, followed by the 0.12 and 0.06 mm^2^ devices. This area dependence is consistent with the expected geometric scaling of distributed conduction in the lateral device geometry. Despite the variation in absolute current, the overall hysteretic switching characteristics remain qualitatively similar across the three electrode areas, with no systematic change in the switching behavior observed within the investigated range. The simultaneous dependence of absolute current on device geometry and preservation of the switching characteristics supports a distributed conduction mechanism rather than a conduction process dominated by a localized, area-insensitive pathway.

**Figure S16. SEM images of the initial Pt/Cu_1-_*_x_*Ag*_x_*I/Ag devices and EDS line scans of Cu, I, Ag, and Pt elements.** Panels (a-e) correspond to functional layers with *x* = 0, 0.024, 0.164, 0.20, and 0.509, respectively.

For the as-fabricated Pt/Cu_1-_*_x_*Ag*_x_*I/Ag devices (*x* = 0, 0.024, 0.164, 0.20, and 0.509), unbiased cross sectional SEM images reveal sharply defined interfaces between the electrodes and the functional layer, with no metal accumulation observed at either the positive or negative electrodes. EDS analysis further confirms that Cu, Ag, and I are uniformly distributed across the functional layer, with no evidence of localized compositional segregation or elemental enrichment, indicating that the devices are free of pre-existing metal accumulation in the pristine state.

**Figure S17. SEM images of Pt/Cu_1-x_Ag_x_I/Ag synaptic devices and EDS line scans of Cu, I, Ag, and Pt after applying a positive bias to the Pt electrode.** Panels (a-e) correspond to functional layers with x = 0, 0.024, 0.164, 0.20, and 0.509, respectively.

In the Pt/Cu_1-_*_x_*Ag*_x_*I/Ag synaptic devices, upon applying a bias of 8-10 V (with Pt as the anode and Ag grounded), pronounced metallic enrichment is consistently observed at the grounded electrode, as shown in Fig. S17. For CuI, a strong Cu signal emerges near the ground side interface, whereas the Ag and I profiles remain nearly unchanged, indicating that the accumulated species originates from Cu⁺ migration. For *x* = 0.024 and 0.088, both Cu and Ag peaks are detected at the accumulation region, showing that Ag^+^ ions also migrate readily through the functional layer. When *x* ≥ 0.164, only Ag is detected at the grounded electrode. Importantly, Ag and I remain uniformly distributed throughout the bulk of the functional layer across all compositions, confirming that the observed metallic clusters arise from field-induced migration of Cu^+^ or Ag^+^ ions followed by their reduction at the grounded interface. The migration and reduction of these mobile ions generate additional V_Cu_ defects and substantially increase the hole concentration within the film, thereby enabling and enhancing the resistive switching behavior.

**Figure S18. SEM images of Pt/Cu_1-_*_x_*Ag*_x_*I/Ag synaptic devices and EDS line scans of Cu, I, Ag, and Pt after applying a reverse bias to the Pt electrode.** Panels (a-e) correspond to functional layers with *x* = 0, 0.024, 0.164, 0.20, and 0.509, respectively.

Upon applying a reverse bias to the Pt/Cu_1-_*_x_*Ag*_x_*I/Ag devices, with the Ag electrode positively biased and Pt grounded, elemental enrichment is observed near the grounded Pt interface (Fig. S18). For CuI (*x* = 0), only Cu is detected near the grounded Pt interface, even when the Ag electrode is positively biased. For *x* = 0.024 and 0.088, both Cu and Ag are detected, whereas only Ag is detected for *x* ≥ 0.164. This composition-dependent evolution of the accumulated species is consistent with the Cu_1-_*_x_*Ag*_x_*I functional-layer composition and supports the functional layer as the primary source of the mobile cations. The I signal remains uniformly distributed throughout the functional layer.

**Figure S19. Morphology and elemental distribution of reduced metal accumulation in Pt/Cu_1-x_Ag_x_I/Ag devices under high bias. SEM images and corresponding EDS mappings of Cu, Ag, and I acquired at the Ag electrode after applying a continuous bias of 8-10 V to Pt/Cu_1-x_Ag_x_I/Ag devices.** (a) CuI, (b) Cu_1-x_Ag_x_I (x = 0.024), (c) Cu_1-x_Ag_x_I (x = 0.088), (d) Cu_1-x_Ag_x_I (x = 0.164), (e) Cu_1-x_Ag_x_I (x = 0.20), and (f) Cu_1-x_Ag_x_I (x = 0.509).

After applying a continuous bias of 8-10 V to Pt/Cu_1-_*_x_*Ag*_x_*I/Ag devices, the microstructural features of the reduced Cu and Ag metals accumulated at the Ag electrode are shown in Fig. S19. The metallic deposits exhibit a loose and discontinuous morphology, suggesting a negligible contribution to interfacial barrier modulation. EDS analysis reveals that in Pt/CuI/Ag devices (Fig. S19a), a pronounced Cu signal is detected at the Ag electrode (cathode), while the Ag signal primarily originates from the Ag electrode itself. With increasing Ag content in the functional layer, the Cu mapping intensity progressively diminishes, accompanied by a concomitant enhancement of the Ag signal. In devices with low Ag concentrations (*x* = 0.024 and 0.088), both Cu and Ag metal accumulations are observed at the cathode side, whereas only Ag metal enrichment is detected when the Ag content exceeds *x* ≥ 0.164. This compositional evolution indicates that Cu^+^ migration is gradually suppressed with increasing Ag incorporation, in good agreement with the EDS line-scan results discussed above.

**Figure S20.** **Electrochemical impedance spectroscopy and ionic conductivity of Cu_1-_*_x_*Ag*_x_*I at different temperatures.** (a-c) Nyquist plots measured at 300, 315 and 330 K, respectively, for Cu_1-_*_x_*Ag*_x_*I with different Ag contents. The solid curves represent fitted data using the (R (QR)) equivalent circuit. (d) Ionic conductivity as a function of Ag content at different temperatures. (e) Arrhenius plots of lnσ_i_ versus 1000/T. (f) Transport activation energy Ea extracted from the Arrhenius fits as a function of Ag content.

To evaluate the effect of Ag incorporation on ionic transport, bulk Cu_1-_*_x_*Ag*_x_*I pellets were characterized by temperature-dependent EIS at 300, 315 and 330 K. As shown in Fig. S20a-c, the inset illustrates the (R(QR)) equivalent circuit employed for fitting using the ZSimpWin package, and the extracted parameters are summarized in Table S2. The extracted conductivity increases with increasing Ag content at all measured temperatures (Fig. S20d). The temperature dependence of the conductivity was further analyzed using the Arrhenius relationship: ^[6]^

σ = σ_0_ exp (-$\frac{E_{a}}{K_{B}T}$)	(S4)

where σ_0_ is the pre-exponential factor, Ea is the activation energy, K_B_ is the Boltzmann constant, and T is the absolute temperature. The corresponding Arrhenius plots of lnσ_i_ versus 1000/T are shown in Fig. S20e. The extracted Ea decreases systematically from approximately 0.51 eV for pristine CuI to 0.46, 0.43, 0.33 and 0.30 eV with increasing Ag contents of 3.9%, 6.9%, 16.7% and 42.2%, respectively (Fig. S20f). This decrease in transport activation energy provides quantitative evidence that Ag incorporation facilitates thermally activated ionic transport by reducing the effective transport barrier.^[7]^

**Figure S21. EDS elemental mappings of the Cu_1-_*_x_*Ag*_x_*I (*x* = 0.088) film interface.** (a-e) Uniform elemental distributions in the pristine (unbiased) state; (f-j) Elemental redistribution after prolonged negative bias applied to the Pt electrode.

To elucidate the ionic transport behavior, EDS was performed on the Pt/Cu_1-_*_x_*Ag*_x_*I (*x* = 0.088) /Si structure in regions free of electrical bias. As shown in Fig. S21(a-e), all constituent elements are uniformly distributed in the pristine state, with no discernible segregation or phase separation, indicating compositional homogeneity and interfacial stability of the as fabricated film. Upon prolonged application of a negative bias to the Pt electrode (Fig. S21(f-j)), a pronounced redistribution of elements is observed. Notably, a distinct accumulation of Cu species emerges at the Pt/Cu_1-_*_x_*Ag*_x_*I (*x* = 0.088) interface (Fig. S21 g), while no corresponding enrichment of I is detected in the same region (Fig. S21 h). This selective enrichment provides compelling evidence that Cu ions undergo directional migration under the applied electric field and are subsequently reduced at the low-potential interface, leading to the formation of metallic Cu. These observations directly confirm that the resistive modulation in Cu_1-_*_x_*Ag*_x_*I-based devices is governed by electric-field-driven Cu ion migration and interfacial redox reactions, rather than conventional CF formation. This mechanism offers critical insight into the fundamental switching physics and provides a solid foundation for further device optimization.

**Figure S22. HR-TEM characterization of the metal-accumulated region after biasing in the *in situ* TEM experiment.** (a) HR-TEM image; (b) corresponding FFT pattern of the nanoparticle in (a); (c) atomic model of fcc Cu viewed along the [110] zone axis.

To further clarify the chemical state of the Cu species accumulated during electrical switching, HR-TEM was performed on the Cu-rich regions identified by EDS after electrical biasing. Well-crystallized nanoparticles with clear lattice fringes are observed. The corresponding FFT pattern shows lattice spacings of approximately 0.208 and 0.181 nm, which are assigned to the (111) and (200) planes of fcc Cu, respectively. The atomic model further corroborates the fcc Cu structure. Together with the EDS evidence for Cu accumulation, these observations support the reduction of migrated Cu^+^ species to metallic Cu^0^. The coupling between cation migration, vacancy formation and conductance modulation can be described using defect chemistry. When Cu^+^ is removed from its normal Cu sublattice site and reduced to neutral Cu^0^, the process can be schematically expressed in Kröger–Vink notation as:

$${Cu}_{Cu}^{\times}\to V_{Cu}^{'}+ {Cu}^{0}+h$$

where $V_{Cu}^{'}$ denotes a negatively charged Cu vacancy and *h* represents the compensating hole. This reaction provides a charge-balanced description of the coupling between Cu^+^ removal, Cu-vacancy formation and the hole population in the p-type Cu_1-_*_x_*Ag*_x_*I lattice. During SET, the applied electric field drives Cu^+^/Ag^+^ redistribution toward the grounded interface, while the accumulated Cu species can undergo local reduction. The associated evolution of the cation/vacancy distribution modifies the local electronic structure and interfacial Schottky barrier, resulting in an increase in conductance. Under reversed bias, the redistributed ionic species partially recover their initial spatial distribution, leading to recovery of the cation/vacancy configuration and interfacial barrier, and consequently to conductance depression.

**Figure S23. Time‑series in‑situ TEM evolution of the Cu_1-_*_x_*Ag*_x_*I (*x* = 0.088) thin film under zero bias.**

Time-series *in situ* TEM images of Cu_1-_*_x_*Ag*_x_*I (*x*=0.088) under zero-bias electron-beam irradiation. The device stack consists of Si/Cu_1-_*_x_*Ag*_x_*I (*x*=0.088)/Pt. The same electron-beam energy and current density as those used in the biased in situ TEM measurements were applied for a total irradiation time of 110 s. Images were acquired at the pristine state and after 20, 50, 80, 100, and 110 s of continuous electron-beam irradiation. No obvious spatial redistribution or interfacial accumulation of Cu/Ag species is observed in the absence of an external electrical bias, and the film remains structurally stable. The images are extracted from Supporting Video S4, shown at 10× playback speed.

**Figure S24. *I-V* cyclic characteristics of Pt/Cu_1-_*_x_*Ag*_x_*I/Ag (*x* = 0, 0.024, 0.088, 0.164, 0.20, and 0.509) devices following two months of storage in a dry box.**

**Figure S25. *I-V* cyclic characteristics of Pt/Cu_1-_*_x_*Ag*_x_*I/Ag (*x* = 0, 0.024, 0.088, 0.164, 0.20, and 0.509) devices following one year of storage in a dry box.**

**Figure S26. *I-V* cyclic characteristics of Pt/Cu_1-x_Ag_x_I/Ag (x = 0, 0.024, 0.088, 0.164, 0.20, and 0.509) devices following two years of storage in a dry box.**

To assess the long-term reliability of Pt/Cu_1-_*_x_*Ag*_x_*I/Ag devices, a 24-month storage stability study was performed. After storage, the same electrical measurement protocol was applied to the identical batch of devices, enabling a systematic comparison of key performance metrics before and after aging. Figs S24-S26 show the *I-V* cycling characteristics of devices stored in a desiccator for 2 months,12 mouths and 24 months, respectively, over five consecutive scans. Remarkably, even after two years of storage, the resistive switching behavior -including ON/OFF ratio and nonvolatility- remains largely unchanged. At a read voltage of 0.35 V, the ON/OFF ratio still exceeds 10^4^. As illustrated in Fig. S26c, devices with *x* = 0.088 maintain the most stable performance throughout five consecutive *I-V* cycles. These findings demonstrate outstanding aging resistance and long-term stability, underscoring the devices’ reliability and practical application potential.

**Figure S27. Resistive switching characteristics of Pt/Cu_1-x_Ag_x_I/Ag devices under different voltage amplitudes.** *I-V* responses of devices with Ag compositions of (a) *x* = 0, (b) *x* = 0.024, (c) *x* = 0.088, (d) *x* = 0.164, (e) *x* = 0.20, and (f) *x* = 0.509.

The dependence of resistive switching on the programming voltage was investigated by varying the voltage window from ±1 V to ±5 V. For all compositions of the synaptic devices, the ON/OFF ratio increases monotonically with the maximum applied voltage. In devices with higher Ag content, the ratio reaches saturation at approximately ±5 V, with no further substantial improvement observed beyond this threshold. This behavior can be attributed to the kinetics of ion migration and the evolution of conductive channels. The amplitude of the applied voltage directly governs the internal electric field strength; higher fields provide stronger driving forces, facilitating the migration of additional defects and ions. This, in turn, promotes the formation of more vacancies within the functional layer, typically resulting in a reduced resistance of the LRS.

**Figure S28. Resistive switching characteristics of Pt/Cu_1-x_Ag_x_I/Ag devices under different scanning rates.** *I-V* responses of devices with Ag compositions of (a) *x* = 0, (b) *x* = 0.024, (c) *x* = 0.088, (d) *x* = 0.164, (e) *x* = 0.20, and (f) *x* = 0.509.

Beyond the influence of voltage amplitude, we investigated the ion tunability of Pt/Cu_1-_*_x_*Ag*_x_*I/Ag synaptic devices. Unlike conventional *I-V* cycling, reducing the voltage sweep rate results in a more pronounced increase in device conductance. Notably, slower sweep rates also enhance the conductance hysteresis, providing further evidence for the activation of ionic transport within the functional layer. ^[8]^

**Figure S29. Long-term retention performance of Pt/Cu_1-x_Ag_x_I/Ag synaptic devices.** (a) *x* = 0, (b) *x* = 0.024, (c) *x* = 0.164, (d) *x* = 0.20, and (e) *x* = 0.509.

We assessed the long-term data retention (nonvolatility) of Pt/Cu_1-_*_x_*Ag*_x_*I/Ag synaptic devices. As shown in Fig. S29, after continuous operation for 4×10^3^ s, the ON/OFF ratio exhibits a strong dependence on composition. Devices with *x* = 0.088 maintain an ON/OFF ratio of approximately 100, whereas other compositions display less pronounced nonvolatility under prolonged testing. In the *x* = 0.088 devices, the moderate introduction of Ag⁺ reduces the overall ion migration barrier, enhancing switching speed and reversibility, thereby achieving an optimal balance between efficient ion transport and stable interfacial regulation. These observations align with recent studies on ionic memristors, where moderate ionic doping is recognized as a key strategy to optimize migration barriers and interface stability, enabling both high ON/OFF ratios and long-term retention. ^[9]^

**Figure S30 Analysis of the nonlinearity in synaptic weight updates based on the data in Fig. 5(d). (a) Absolute conductance change (|ΔG|) upon successive pulses. (b) Non‑linearity analysis of LTP-LTD weight‑update curves.**

To quantitatively evaluate the synaptic weight-update characteristics, we analyzed the asymmetric nonlinearity between LTP and LTD, the nonlinearity of the potentiation and depression processes. he symmetry between potentiation and depression was quantitatively evaluated using the asymmetric nonlinearity factor (*ANF*). *ANF* was calculated from the experimental LTP and LTD weight-update characteristics according to: ^[10, 11]^

*ANF* = $\frac{G_{LTP}(\frac{n}{2})-G_{LTD}(\frac{n}{2})}{G_{max}-G_{min}}$	(1)

here, *G_LTP_* ($\frac{n}{2}$) and *G_LTD_* ($\frac{n}{2}$) are the intermediate conductance values of LTP and LTD, respectively, *G_max_* and *G_min_* are the maximum and minimum conductance of the device. Using the experimental LTP and LTD characteristics in Fig. 5d, the calculated asymmetric nonlinearity factor is *ANF* = 0.34. A smaller *ANF* indicates a smaller asymmetry between the potentiation and depression characteristics. Additionally, we calculated the rate of conductance change between adjacent pulses for the LTP and LTD processes, as illustrated in Fig. S30 a. It can be clearly observed that the conductance (*ΔG*) increases with the increasing number of pulses during LTP, whereas it decreases during LTD. This behavior reveals that the device exhibits inherent nonlinear conductance modulation. The nonlinear evolution of the individual LTP and LTD processes was further quantified by fitting the experimental weight-update characteristics. The conductance evolution during potentiation and depression was described by: ^[10, 12-15]^

*G*_LTP_ = *G*_min_ + *B* · (1-$e^{-\frac{P}{A}}$)	(2)

*G*_LTD_ = *G*_max_ - *B* · (1-$e^{\frac{{(P-P}_{max})}{A}}$)	(3)

*B* = $\frac{G_{max}-G_{min}}{1-e^{\frac{-P_{max}}{A}}}$	(4)

α = $\frac{1.726}{A + 0.162}$	(5)

where *G*_max_ is the maximum conductance, *G*_min_ denotes the minimum conductance, and *P*_max_ denotes the maximum number of pulses required to tune the device from the minimum to maximum conductance state. *A* is the parameter that determines the weight update's nonlinearity behavior, *B* is simply a function of *A*, and *α* is a parameter characterizing the nonlinearity. The fitting results are demonstrated in Fig. S30 b; the nonlinearity parameter of Pt/Cu_1-_*_x_*Ag*_x_*I/Ag (x = 0.088) memristor is -3.10 for LTP and 7.98 for LTD, respectively. The relatively different α values indicate that the potentiation and depression processes exhibit distinct update nonlinearities under the applied pulse conditions. The asymmetry is calculated by the difference in the absolute value of the potentiation and depression nonlinearity: ^[16]^

||α_LTP_-|α_LTD_|| (6)

The asymmetry is determined to be 4.88.

**Figure S31 Conductance modulation characteristics and cycle-to-cycle/device-to-device uniformity of synaptic devices. (a) Four consecutive LTP/LTD cycles of a single device; (b) Average LTP/LTD curves and corresponding standard deviation (SD) extracted from (a); (c) Conductance responses of five different devices under 50 potentiation and 50 depression pulses, showing device-to-device variations; (d) Average LTP/LTD curves and SD extracted from (c).**

Cycle-to-cycle (C2C) and device-to-device (D2D) variations were quantified to evaluate the reproducibility of the synaptic weight-update characteristics. C2C variation was evaluated from repeated programming cycles performed on the same device, whereas D2D variation was evaluated using five independently measured devices. For each programming pulse, the mean and standard deviation were calculated from the corresponding repeated cycles or independently measured devices. The mean represents the average weight-update response, while the standard deviation characterizes the corresponding dispersion. The mean ± standard deviation profiles are shown as the central line and shaded region, respectively. The coefficient of variation (CV) was calculated as:

CV = $\frac{\sigma}{\mu}\times100\%$	(S7)

where μ and σ denote the mean and standard deviation, respectively. The resulting CVs are 67.0% and 50.3% for C2C LTP and LTD, respectively, and 10.0% and 33.2% for D2D LTP and LTD, respectively.

The number of experimentally distinguishable conductance levels was evaluated using a 3σ criterion. ^[17]^ Here, σ represents the standard deviation of the device-to-device conductance variation obtained from five independently measured devices. A new conductance level was considered distinguishable when the cumulative conductance change from the preceding level exceeded 3σ. The analysis was performed sequentially along the programming-pulse sequence. Specifically, the cumulative conductance change over a given pulse interval was compared with the 3σ threshold. When the cumulative change exceeded 3σ, the corresponding conductance evolution was regarded as experimentally distinguishable from the preceding level. For LTP, eight distinguishable conductance levels were identified. The corresponding cumulative pulse intervals were 0-5, 6-7, 8-10, 11-15, 16-21, 22-29, 30-39 and 40-50. For LTD, five distinguishable conductance levels were identified over the corresponding pulse intervals of 51-52, 53-54, 55-61, 62-79 and 80-100.

**Figure S32. Spike number-dependent plasticity (SNDP) simulation of Pt/Cu_1-x_Ag_x_I/Ag (*x* = 0.088) synaptic devices under varying numbers of voltage pulses. (a) postsynaptic current, (b) SNDP**

Pt/Cu_1-_*_x_*Ag*_x_*I/Ag (*x* = 0.088) synaptic devices were stimulated with varying numbers of voltage pulses, each with an amplitude of 5 V, a width of 50 ms, and an interval of 20 ms. As shown in Fig. S32, the devices exhibit spike-number-dependent plasticity (SNDP), with the postsynaptic current (PSC) increasing as the number of pulses grows. To quantitatively compare PSC variations under different pulse-number stimulations, we extracted the PSC values following the first (A_1_) and N-times (A_N_) pulses, and used the ratio A_N_/A_1_ to represent changes in synaptic weight. The A_N_/A_1_ ratio increases markedly with increasing pulse number. Notably, when 80 pulses were applied, the SNDP ratio (A_80_/A_1_) exceeded 7200%, 46 times higher than that observed with only 10 pulses (Fig. S32b).

**Figure S33. Emulation of spike-dependent synaptic plasticity in Pt/Cu_1-x_Ag_x_I/Ag (*x* = 0.088) synaptic devices under different pulse conditions. (a) (SVDP), (b) (SWDP), and (c) (SRDP) characteristics measured under varying pulse amplitudes, pulse widths, and pulse frequencies, respectively.**

In biological synapses, synaptic weight modulation depends on spike amplitude, spike width, and spike frequency. ^[18, 19]^ To emulate spike-voltage-dependent plasticity (SVDP), we applied sequences of voltage pulses with varying amplitudes to the synaptic devices, using a pulse width of 100 ms and a pulse interval of 10 ms. As shown in Fig. S33a, for a sequence of five pulses, the PSC increases with pulse amplitude, and an increased number of pulses in the sequence further enhances the PSC. Furthermore, spike-width-dependent plasticity (SWDP, Fig. S33b) and spike-rate-dependent plasticity (SRDP, Fig. S33c) were realized by modulating the pulse width and frequency, respectively. These findings confirm that the fabricated Pt/Cu_1-_*_x_*Ag*_x_*I/Ag (*x* = 0.088) devices are capable of emulating the essential functions of biological synapses.

**Figure S34. Schematic illustration of a three-layer artificial neural network for digit recognition.**

The network comprises an input layer, a hidden layer, and an output layer, consisting of 784 (28×28) input neurons, 100 hidden neurons, and 10 output neurons corresponding to digits 0-9. Pt/Cu_1-_*_x_*Ag*_x_*I/Ag (*x* = 0.088) synaptic devices serve as the storage elements within the array. During training, each pixel of the MNIST pattern is sequentially fed into the input neurons, processed through the synaptic weight matrix, and propagated to the hidden layer. The synaptic weights are updated via a backpropagation learning algorithm, based on the difference between the outputs of the 10 output neurons and the label of the input MNIST pattern. ^[20, 21]^

**Table S1. Electrical properties of Cu_1-_*_x_*Ag*_x_*I thin films measured at room temperature: carrier concentration (P), conductivity (σ), mobility (μ; positive and negative values indicate p-type and n-type conduction, respectively), and sheet resistance (Rs).**

| **Ag (at%)** | **P (×10^17^ cm^3^)** | **σ (S·cm^-1^)** | **μ (cm^2^·v^-1^·s^-1^)** | **R_S_ (×10^3^ Ω/□)** |
| --- | --- | --- | --- | --- |
| CuI | **378.3** | **36.0** | **5.94** | **2.95** |
| 2.4% Ag | **121.5** | **16.8** | **8.63** | **6.38** |
| 8.8% Ag | **61.1** | **9.19** | **9.38** | **10.28** |
| 16.4% Ag | **12.3** | **0.66** | **3.34** | **141** |
| 20.0% Ag | **6.3** | **0.14** | **1.43** | **700.5** |
| 50.9% Ag | **1.58** | **0.03** | **1.24** | **2566** |

As summarized in Table S1, the incorporation of Ag⁺ (from n-type AgI) into CuI thin films induces electron-hole recombination, leading to a significant reduction in carrier concentration with increasing Ag⁺ content. ^[3]^ At 50.9% Ag⁺ doping, the carrier concentration is modulated by two orders of magnitude (from 10^17^ to 10^19^ cm^-3^), accompanied by a decrease in conductivity (from 36.0 to 0.03 S·cm^-1^) and an increase in sheet resistance. The carrier mobility of Cu_1-_*_x_*Ag*_x_*I thin films exhibits a non-monotonic dependence on Ag doping concentration, initially increasing before decreasing, with the optimal mobility (9.38 cm^2^·V^-1^·s^-1^) achieved at an Ag doping level of 8.8%. This phenomenon can be attributed to the following mechanisms: At the optimal doping concentration (8.8% Ag), the crystallinity of the film is significantly enhanced (Fig 5S), accompanied by improved lattice integrity and reduced defect density, thereby effectively mitigating carrier scattering mechanisms such as grain boundary scattering and ionized impurity scattering. The enhanced mobility plays a pivotal role in improving the performance of memristive devices. First, higher carrier mobility facilitates more efficient charge transport during switching operations, enabling faster device response. Second, the improved mobility reduces Joule heating during device operation, thereby enhancing the reproducibility and stability of resistive switching behavior. Furthermore, superior crystallinity suppresses defect-assisted leakage paths, which is critical for achieving a high ON/OFF ratio (R_ON_/R_OFF_) and robust endurance. In contrast, either insufficient or excessive Ag doping leads to microstructural degradation—insufficient doping fails to adequately improve crystallinity, while excessive doping introduces excessive lattice distortion and defect states, both of which ultimately result in reduced mobility. Notably, the consistently positive mobility values confirm that all films retain p-type (hole) conduction behavior.

**Table. S2. Electrochemical impedance spectroscopy (EIS) fitting parameters of Cu_1-_*_x_*Ag*_x_*I materials at room temperature. The units of T, R and Q are K, Ω and F, respectively.**

| **T (K)** | **Ag (at%)** | **R_0_ (Ω)** | **CPE (Q)** | **n** | **R_1_ (Ω)** | **C (F)** | **Chi-squared** |
| --- | --- | --- | --- | --- | --- | --- | --- |
| 300 | CuI | 535 | 3.38E-11 | 1 | 2.21E+05 | 3.38E-11 | 0.007265 |
|  | 3.9% Ag | 578.1 | 3.36E-11 | 1 | 1.60E+05 | 3.36E-11 | 0.008108 |
|  | 6.9% Ag | 789.7 | 3.70E-11 | 0.9881 | 9.56E+04 | 3.18E-11 | 0.003725 |
|  | 16.7% Ag | 550.3 | 4.85E-11 | 0.978 | 3.82E+04 | 3.61E-11 | 0.001958 |
|  | 42.2% Ag | 446.6 | 5.16E-11 | 0.9739 | 2.90E+04 | 3.60E-11 | 0.001068 |
| 315 | CuI | 530.5 | 4.53E-11 | 0.9808 | 8.14E+04 | 3.54E-11 | 0.003773 |
|  | 3.9% Ag | 535.6 | 3.15E-11 | 0.9997 | 7.97E+04 | 3.14E-11 | 0.008741 |
|  | 6.9% Ag | 482.7 | 6.26E-11 | 0.9463 | 3.89E+04 | 3.00E-11 | 0.001481 |
|  | 16.7% Ag | 458.2 | 4.55E-11 | 0.978 | 3.18E+04 | 3.36E-11 | 0.002915 |
|  | 42.2% Ag | 466.6 | 3.24E-11 | 1 | 1.74E+04 | 3.24E-11 | 0.006711 |
| 330 | CuI | 423.6 | 5.30E-11 | 1 | 3.67E+04 | 5.30E-11 | 0.005056 |
|  | 3.9% Ag | 584.3 | 3.61E-11 | 1 | 3.20E+04 | 3.61E-11 | 0.003391 |
|  | 6.9% Ag | 523.2 | 1.67E-11 | 1 | 2.12E+04 | 1.67E-11 | 0.06698 |
|  | 16.7% Ag | 550.4 | 3.29E-11 | 0.9998 | 1.17E+04 | 3.01E-11 | 0.0265 |
|  | 42.2% Ag | 540.3 | 3.01E-11 | 1 | 9991 | 3.28E-11 | 0.001451 |

The high-frequency intercept on the Z’ axis represents the ohmic resistance R₀ arising from both ionic and electronic transport, whereas R₁ corresponds to the charge-transfer resistance associated with the semicircular arc at the electrode interface ^[22]^. The fitting curves were obtained using the R_0_(Q_1_R_1_) equivalent circuit model in ZSimpWin, where T is absolute temperature, R denotes resistance and Q (CPE) denotes the constant phase element of a non-ideal capacitor. ^[23-25]^ The capacitance for each process can be calculated as^[26]^:

|  | C = $\frac{{(R\times Q)}^{\frac{1}{n}}}{R}$ | （S4） |
| --- | --- | --- |

Here, n represents the similarity between the CPE and an ideal capacitor; when n = 1, the CPE can be considered an ideal capacitor ^[7, 24]^. Fitting data indicate that the ohmic resistance of bulk Cu_1-_*_x_*Ag*_x_*I ranges from 400 to 800 Ω, attributed to the intrinsic conductivity of CuI. Using the R₁ values and the above equation, the ionic conductivity and activation energy of bulk Cu_1-_*_x_*Ag*_x_*I at 300-330 K was determined (Fig. S19). Furthermore, the n values are equal to or very close to 1, demonstrating that the prepared materials exhibit capacitor-like behavior with respect to ionic migration. Notably, the final column of the fitting data reports the error factor, which lies between 10^-2^ and 10^-3^, confirming the accuracy and reliability of the fits.

**Table S3. Benchmark comparison of representative memristive devices and the present device.**

| Device | Pulse | Conductance states | Retention (s) | Asymmetry | On/Off ratio | Energy (nJ) | CV | Shelf-life (days) | Ref. |
| --- | --- | --- | --- | --- | --- | --- | --- | --- | --- |
| Cu/SiC/W | 1.2 V, 60 μs  -1.2 V, 60 μs | 40 | 1×10^3^ | 0.23 | ＞10 | - | 1.15% (LTP and LTD) | - | ^[27]^ |
| Ag/ZnPS_3_/Au | 0.7 V, 10 ns | 256 | 3.6×10^4^ | - | 10^7^ | 1.43×10^-7^ | ＜30% (D2D) | - | ^[28]^ |
| TiN/ETML/HfO*_x_*/TiN | 1.6 V, 50 ns  1.5 V, 50 ns | 128 | 600 (125℃) | 0.59 | 10 | 1.28×10^-3^ | - | - | ^[29]^ |
| TiN/TEL/HfO*_x_* /TiN | 1.3 V, 50 ns  1.5 V, 50 ns | 128 | 3×10^3^ (150℃) | 2.3 | 10 | 1.45×10^-2^ | - | - | ^[30]^ |
| Al/Mo/PCMO/Pt | 2.5 - 4.5 V  1μs - 10 ms | 100 | 10^4^ (120℃) | 1.99 | ~10^3^ | 6.84×10^-2^ | - | - | ^[31]^ |
| Pt/AlO*_x_*/TiN /PCMO/Pt | -3 V, 1ms  3 V, 1ms | 50 | 10^4^ | 3.08 | - | 3.91×10^-1^ | - | - | ^[32]^ |
| Al/AlO*_x_*/HfO_2_/Ti/TiN | 0.9 V, 100 μs  -1 V, 100 μs | 40 | - | 0.75 | ~10^2^ * | 5.92 | - | - | ^[33]^ |
| Ti/AlO*_x_*/TaO*_x_*/Pt | 0.85 V, 5 μs  -1.15 V, 5 μs | 80 | 10^4^ | 2.15 | - | 18.9 | - | - | ^[34]^ |
| Ti/PdSeO_x_/PdSe_2_/Au | 0.9 V, 50 μs  -0.9 V, 50 μs | 100 | 8.6×10^5^ | - | ~3×10^2^ | 9×10^-4^ | -3.6%  4.8% | 180 | ^[35]^ |
| ITO/CsCu_2_I_3_/Au | -2 V, 7 ms  2 V, 10 ms | 50 | 40 | 0.5 | 10^2^ | - | - | 160 | ^[36]^ |
| Au/BDAMA_2_Pb_3_I_10_/Au | -1.5 V, 500 μs  1.5 V, 500 μs | 50 | 10^4^ | 0.0005 | - | 2.81×10^-3^ | - | 210 | ^[16]^ |
| Pt/Cu_1-_*_x_*Ag*_x_*I/Ag  *x* = 0.088 | 5 V, 100 ms  -5 V, 10 ms | 50 | 4×10^3^ | 4.88 | ＞10^5^ | 14 .1 | 67.0% (C2C_LTP_)  50.3% (C2C_LTD_)  10.0% (D2D_LTP_)  33.2% (D2D_LTD_) | 730 | This work |

**References**

[1]	TAREY R D, RAJU T A. A method for the deposition of transparent conducting thin films of tin oxide [J]. Thin Solid Films, 1985, 128(3-4): 181-9.

[2]	YANG C, SOUCHAY D, KNEIß M, et al. Transparent flexible thermoelectric material based on non-toxic earth-abundant p-type copper iodide thin film [J]. Nature Communications, 2017, 8(1).

[3]	ANNADI A, GONG H. Success in both p-type and n-type of a novel transparent AgCuI alloy semiconductor system for homojunction devices [J]. Applied Materials Today, 2020, 20: 100703.

[4]	MAZUMDAR D, GOVINDACHARYULU P A, BOSE D N. Electronic conductivity of AgI using D.C. polarization and charge transfer techniques [J]. Journal of Physics and Chemistry of Solids, 1981, 43(9): 933-40.

[5]	YANG C, KNEIß M, SCHEIN F-L, et al. Room-temperature Domain-epitaxy of Copper Iodide Thin Films for Transparent CuI/ZnO Heterojunctions with High Rectification Ratios Larger than 10^9^ [J]. Scientific Reports, 2016, 6(1).

[6]	FENG L, AN S, WANG X, et al. Synthesis of ZIF-62 Glass and Solid-state Lithium Superconductor Composite Membranes for Electrodialytic Lithium Extraction [J]. Nature Communications, 2026, 17(1): 7414.

[7]	OUYANG Y, ZHU D, ZHU C, et al. Composite electrolyte with Ruddlesden-Popper structure Sm_1.2_Sr_0.8_Ni_0.6_Fe_0.4_O_4+δ_ for high-performance low temperature solid oxide fuel cells [J]. International Journal of Hydrogen Energy, 2023, 48(1): 268-79.

[8]	CHENG X, ZHONG Z, ZHUANG Y, et al. High Precision Conductance Modulation in CuCrP_2_S_6_ Synaptic Devices for Enhanced Neuromorphic Computing [J]. Advanced Functional Materials, 2025, 35(42).

[9]	ZHONG Z, CHENG X, SONG W, et al. Reconfigurable logic circuits and rectifier based on two-terminal ionic homojunctions [J]. Device, 2025, 3(5).

[10]	PAN J, KAN H, LIU Z, et al. Flexible TiO_2_-WO_3-x_ hybrid memristor with enhanced linearity and synaptic plasticity for precise weight tuning in neuromorphic computing [J]. npj Flexible Electronics, 2024, 8(1): 70.

[11]	KRISHNAPRASAD A, DEV D, SHAWKAT M S, et al. Graphene/MoS_2_/SiO_x_ memristive synapses for linear weight update [J]. npj 2D Materials and Applications, 2023, 7(1): 22.

[12]	WANG C, MAO G Q, HUANG M, et al. HfO(x) /AlO(y) Superlattice-Like Memristive Synapse [J]. Adv Sci (Weinh), 2022, 9(21): e2201446.

[13]	CHEN Y, ZHOU Y, ZHUGE F, et al. Graphene-ferroelectric transistors as complementary synapses for supervised learning in spiking neural network [J]. npj 2D Materials and Applications, 2019, 3(1): 31.

[14]	MALLIK S, TERABE K, TSURUOKA T. Sub-Millisecond and Energy-Efficient Electrochemical Synaptic Transistors with a Partially Reduced Graphene Oxide Channel [J]. ACS Applied Materials & Interfaces, 2025, 17(17): 25674-83.

[15]	TANG J, HE C, TANG J, et al. A Reliable All-2D Materials Artificial Synapse for High Energy-Efficient Neuromorphic Computing [J]. Advanced Functional Materials, 2021, 31(27): 2011083.

[16]	KIM S J, IM I H, BAEK J H, et al. Linearly programmable two-dimensional halide perovskite memristor arrays for neuromorphic computing [J]. Nat Nanotechnol, 2025, 20(1): 83-92.

[17]	CAI Y, YANG J, HOU Y, et al. 8-bit states in 2D floating-gate memories using gate-injection mode for large-scale convolutional neural networks [J]. Nature Communications, 2025, 16(1): 2649.

[18]	VAN DE BURGT Y, LUBBERMAN E, FULLER E J, et al. A non-volatile organic electrochemical device as a low-voltage artificial synapse for neuromorphic computing [J]. Nature Materials, 2017, 16(4): 414-8.

[19]	LAO J, XU W, JIANG C, et al. An air-stable artificial synapse based on a lead-free double perovskite Cs2AgBiBr6 film for neuromorphic computing [J]. Journal of Materials Chemistry C, 2021, 9(17): 5706-12.

[20]	FENG G, ZHU Q, LIU X, et al. A ferroelectric fin diode for robust non-volatile memory [J]. Nat Commun, 2024, 15(1): 513.

[21]	LAO J, YAN M, TIAN B, et al. Ultralow‐Power Machine Vision with Self‐Powered Sensor Reservoir [J]. Advanced Science, 2022, 9(15).

[22]	RAUF S, ZHU B, SHAH M A K Y, et al. Tailoring triple charge conduction in BaCo_0.2_Fe_0.1_Ce_0.2_Tm_0.1_Zr_0.3_Y_0.1_O_3−δ_ semiconductor electrolyte for boosting solid oxide fuel cell performance [J]. Renewable Energy, 2021, 172: 336-49.

[23]	ZHU B, HUANG Y, FAN L, et al. Novel fuel cell with nanocomposite functional layer designed by perovskite solar cell principle [J]. Nano Energy, 2016, 19: 156-64.

[24]	LIU K, GANESH K S, NIE J, et al. Characterizing the Blocking Electron Ability of the Schottky Junction in SnO_2_-SDC Semiconductor–Ionic Membrane Fuel Cells [J]. ACS Sustainable Chemistry & Engineering, 2020, 8(28): 10357-68.

[25]	WANG B, ZHU B, YUN S, et al. Fast ionic conduction in semiconductor CeO_2-δ_ electrolyte fuel cells [J]. NPG Asia Materials, 2019, 11(1).

[26]	LU Y, AKBAR M, XIA C, et al. Catalytic membrane with high ion–electron conduction made of strongly correlated perovskite LaNiO_3_ and Ce_0.8_Sm_0.2_O_2-δ_ for fuel cells [J]. Journal of Catalysis, 2020, 386: 117-25.

[27]	LIU Q, KAPUR O, DING B, et al. Linear and Programmable Long-Term Plasticity in PECVD Amorphous SiC Memristors [J]. Advanced Electronic Materials, 2026, n/a(n/a): e70508.

[28]	JI Y, WANG L, LONG Y, et al. Ultralow energy adaptive neuromorphic computing using reconfigurable zinc phosphorus trisulfide memristors [J]. Nature Communications, 2025, 16(1): 6899.

[29]	WU W, WU H, GAO B, et al. A Methodology to Improve Linearity of Analog RRAM for Neuromorphic Computing; proceedings of the 2018 IEEE Symposium on VLSI Technology, F 18-22 June 2018, 2018 [C].

[30]	WU W, WU H, GAO B, et al. Improving Analog Switching in HfOx-Based Resistive Memory With a Thermal Enhanced Layer [J]. IEEE Electron Device Letters, 2017, 38(8): 1019-22.

[31]	MOON K, FUMAROLA A, SIDLER S, et al. Bidirectional Non-Filamentary RRAM as an Analog Neuromorphic Synapse, Part I: Al/Mo/Pr_0.7_Ca_0.3_MnO_3_ Material Improvements and Device Measurements [J]. IEEE Journal of the Electron Devices Society, 2018, 6: 146-55.

[32]	PARK S, SHERI A, KIM J, et al. Neuromorphic speech systems using advanced ReRAM-based synapse; proceedings of the 2013 IEEE International Electron Devices Meeting, F, 2013 [C]. IEEE.

[33]	WOO J, MOON K, SONG J, et al. Improved Synaptic Behavior Under Identical Pulses Using AlOx/HfO_2_ Bilayer RRAM Array for Neuromorphic Systems [J]. IEEE Electron Device Letters, 2016, 37(8): 994-7.

[34]	SUN Y, XU H, WANG C, et al. A Ti/AlOx/TaOx/Pt Analog Synapse for Memristive Neural Network [J]. IEEE Electron Device Letters, 2018, 39(9): 1298-301.

[35]	LI Y, CHEN S, YU Z, et al. In-Memory Computing using Memristor Arrays with Ultrathin 2D PdSeO_(x_) /PdSe_(2)_ Heterostructure [J]. Adv Mater, 2022, 34(26): e2201488.

[36]	KWAK K J, BAEK J H, LEE D E, et al. Ambient Stable All Inorganic CsCu_(2)_I_(3)_ Artificial Synapses for Neurocomputing [J]. Nano Lett, 2022, 22(14): 6010-7.
